# Evolutionary Integration and Glucocorticoid Regulation of the Respiratory System: Structure, Function, and Homeostatic Adaptation

**DOI:** 10.3390/medsci14010090

**Published:** 2026-02-14

**Authors:** Gianfranco Umberto Meduri, Antoni Torres

**Affiliations:** 1 Department of Medicine and Pharmaceutical Sciences, University of Tennessee Health Science Center, Memphis, TN 38163, USA; 2 Institut d’Investigacions Biomédiques August Pi i Sunyer (IDIBAPS), University of Barcelona, 08036 Barcelona, Spain; 3 Centro de Investigación Biomédica en Red de Enfermedades Respiratorias (CIBERES), 28029 Madrid, Spain

**Keywords:** glucocorticoid receptor alpha (GRα), evolutionary physiology, respiratory homeostasis, lung development and evolution, mitochondrial bioenergetics, immune–metabolic integration

## Abstract

The vertebrate respiratory system arose under evolutionary pressures that linked increasing atmospheric oxygen levels to the metabolic demands of mitochondria. This transition—from ancestral gill-based exchange to the highly alveolated mammalian lung—was accompanied by the emergence of a hormonal regulatory axis centered on the glucocorticoid receptor alpha (GRα). Over time, GRα became deeply integrated into the architecture and function of the respiratory system, aligning pulmonary performance with organismal homeostasis across different developmental stages, environmental challenges, and disease states. This review combines evolutionary, embryological, and molecular evidence to explain how GRα shapes respiratory structure and function. We trace the evolution from ancient oxygen-sensing systems to mammalian alveoli and endothelial adaptations, demonstrating how conserved developmental pathways (including WNT, FGF, BMP, and SHH) are repurposed during both organogenesis and repair. Genetic models show that GRα is essential for preparing the lung for postnatal life, coordinating the reciprocal signaling between mesenchyme and epithelium that drives branching, septation, extracellular matrix organization, and the development of functional alveolar units. In the mature lung, GRα maintains the stability of the alveolar–capillary interface and coordinates immune, vascular, and metabolic functions to support efficient gas exchange. Its actions also extend to red blood cell biology and the regulation of stress erythropoiesis, linking pulmonary oxygen management with systemic oxygen delivery. Mechanistically, GRα interacts with circadian and hypoxia pathways and activates mitochondrial programs that enhance energy production and redox homeostasis during stress. By integrating these regulatory layers across developmental and physiological contexts, this review reframes GRα not simply as a stress-response receptor but as a non-redundant system-level integrator of respiratory homeostasis. Understanding this layered control not only explains the benefits of antenatal corticosteroids but also highlights the therapeutic value of phase-specific, precision modulation of the GC–GRα axis—along with strategies that support GC–GR signaling—to reestablishing and maintaining homeostasis in acute and chronic pulmonary disorders.

## 1. Introduction

The vertebrate respiratory system is among the most structurally complex and functionally dynamic organ systems in biology, shaped by evolutionary pressures to meet the escalating bioenergetic demands of multicellular organisms. As atmospheric oxygen levels rose and mitochondria became central to cellular metabolism, selective pressures favored the development of increasingly specialized gas-exchange structures. In terrestrial vertebrates, this culminated in the highly organized architecture of the lungs—comprising branching airways, delicate alveolar sacs, and an expansive capillary network—designed to maximize oxygen uptake and eliminate carbon dioxide.

Embedded within this respiratory machinery is an evolutionarily conserved hormonal control axis, centered on the glucocorticoid receptor alpha (GRα). Rather than functioning solely as an acute stress-response receptor, GRα shapes developmental programs and day-to-day pulmonary physiology. It influences epithelial lineage specification, surfactant system maturation, barrier and immune tone, vascular function, and mitochondrial adaptation—allowing the respiratory system to dynamically adjust to developmental cues, environmental fluctuations, and systemic stress. Through this layered integration, the GC–GRα system has contributed to the evolutionary refinement of lung structure and function, enabling coordination between pulmonary physiology and the broader organ systems that maintain whole-body homeostasis.

This manuscript offers a comprehensive review of the evolutionary, developmental, and molecular foundations of pulmonary structure and function, with particular focus on GRα-mediated regulation. Section 1 examines the origins of respiratory adaptations shaped by shifting oxygen availability. Section 2 outlines the emergence of glucocorticoid signaling and its integration into vertebrate strategies for meeting respiratory demands. Section 3 delineates GRα’s indispensable role in fetal lung maturation. Section 4 analyzes the anatomical complexity and functional integration of the adult pulmonary system. Finally, Section 5 synthesizes how GC–GRα signaling coordinates respiratory physiology across multiple subsystems to sustain efficient gas exchange, preserve barrier integrity, and maintain immune and metabolic homeostasis.

## 2. Evolutionary and Embryological Origins of the Respiratory System: Linking Bioenergetic Demand to Structural Innovation

### 2.1. Bioenergetic Pressures and the Need for Oxygen Exchange

The evolution of the vertebrate respiratory system is fundamentally tied to the progressive rise in atmospheric oxygen (O_2_) and the emergence of mitochondria as the central engines of cellular metabolism. Following the Great Oxygenation Event approximately 2.4 billion years ago, eukaryotic cells (cells that contain a true nucleus and membrane-bound organelles, distinguishing them from simpler prokaryotic cells) acquired mitochondria through endosymbiosis (one cell living inside another and becoming an organelle), enabling aerobic respiration and a dramatic expansion in ATP-generating capacity. This transformative bioenergetic shift imposed selective pressure on emerging multicellular organisms to develop increasingly sophisticated mechanisms for acquiring, transporting, and regulating oxygen delivery—particularly to tissues with high mitochondrial density and oxidative demand.

**Figure 1:**
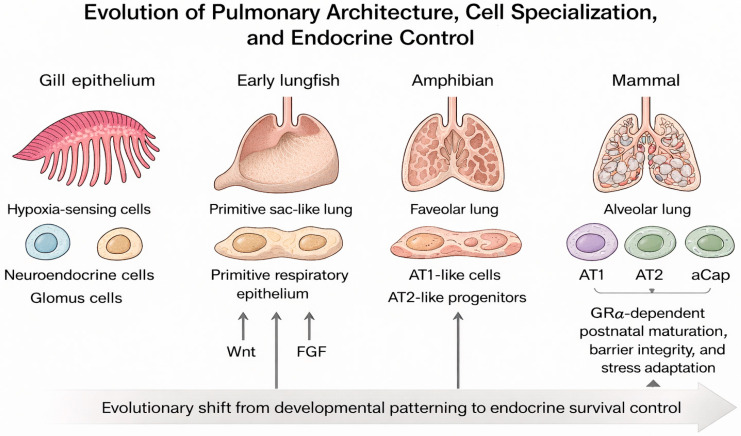
**Evolution of Pulmonary Architecture, Cell Specialization, and Endocrine Control**. **Legend**: The figure illustrates the evolutionary refinement of vertebrate respiratory systems from aquatic gill-based gas exchange to the highly specialized mammalian lung. The ancestral gill epithelium constitutes the earliest oxygen-sensing surface and contains hypoxia-sensing, neuroendocrine, and glomus-like cells that form the evolutionary substrate for later chemosensory mechanisms. In early lungfish, the emergence of a primitive, sac-like lung provided supplemental air breathing but lacked alveolar architecture and discrete alveolar epithelial cell types; lung development at this stage is guided predominantly by conserved Wnt and FGF signaling pathways. Amphibians represent a transitional state, with faveolar lungs showing increased internal compartmentalization and early epithelial diversification, including AT1-like cells and AT2-like progenitors, yet without the thin diffusion barrier characteristic of mammalian alveoli. Mammalian lungs exhibit extensive airway branching, alveolarization, and dense vascular integration, with fully differentiated alveolar epithelial cells (AT1 and AT2) and specialized pulmonary capillary endothelial subtypes (aCap and gCap) that together optimize gas exchange, barrier function, and immune surveillance. This progression reflects an evolutionary shift from local developmental signaling toward endocrine-integrated survival control, with glucocorticoid receptor-α (GRα) emerging as a key regulator of postnatal lung maturation, stress responsiveness, and long-term pulmonary homeostasis, enabling negative-pressure ventilation, endothermy, and sustained aerobic metabolism (*the authors acknowledge ChatGPT 5.2 ’s assistance in creating this figure*).

As a result, the respiratory system evolved not simply as a passive conduit for gas diffusion but as a dynamic, highly regulated network designed to match oxygen availability with the metabolic needs of complex cellular systems. This bioenergetic framework established the foundation for the structural and functional innovations that characterize the vertebrate lung (Figure 1, Evolution of Pulmonary Architecture, Cell Specialization, and Endocrine Control).

### 2.2. From Gills to Lungs: Evolutionary Transitions in Vertebrate Respiration

**Figure 2:**
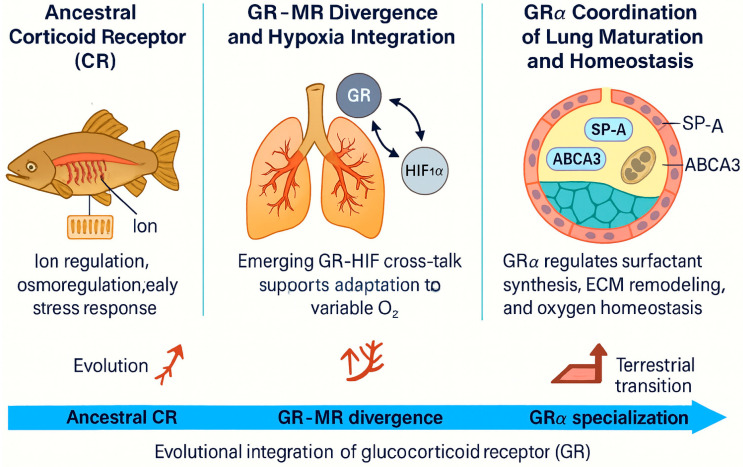
**Evolutionary Integration of Glucocorticoid Receptor (GR) Signaling**. **Legend**: The figure depicts the progressive evolution of GRα-mediated corticosteroid receptor signaling from aquatic to terrestrial vertebrates. The ancestral corticoid receptor (CR) in early fish species primarily mediated ion regulation, osmoregulation, and early stress responses essential for aquatic adaptation. During vertebrate evolution, the divergence of glucocorticoid (GR) and mineralocorticoid (MR) receptors enabled specialized control of systemic homeostasis, energy metabolism, and electrolyte balance. The emerging functional crosstalk between GR and hypoxia-inducible factor 1α (HIF1α) facilitated adaptation to variable oxygen environments during the water-to-land transition. In mammals, the progressive specialization of GRα signaling supported lung maturation by regulating surfactant protein A (SP-A) and ATP-binding cassette transporter A3 (ABCA3), as well as extracellular matrix remodeling, vascular development, and oxygen homeostasis. These integrated functions established GRα as the central regulatory hub for respiratory development, stress adaptation, and postnatal homeostasis (*the authors acknowledge ChatGPT 5.2’s assistance in creating this figure*).

The transition from water to land required profound respiratory innovations and adaptive restructuring of gas-exchange mechanisms. Over roughly 400 million years, vertebrates evolved from gill-based to lung-based respiration, enabling survival in variable oxygen environments and the colonization of terrestrial ecosystems (Figure 1, Evolution of respiratory cells). During this transition, hormonal and molecular regulatory systems—including glucocorticoid signaling—co-evolved with respiratory structures, forming an increasingly integrated physiological axis (Figure 2, Evolutionary Integration of the Glucocorticoid Receptor).

Gills, which evolved before the last common ancestor of vertebrates, initially functioned in ion exchange and acid–base regulation and only later adapted for oxygen uptake through countercurrent gas exchange [1,2,3]. Lungs subsequently appeared as unpaired outpouchings of the foregut in early bony fishes (Osteichthyes) and later evolved into paired organs in tetrapods, significantly improving respiratory efficiency [4,5,6]. In ray-finned fishes, the swim bladder represents a modified lung, highlighting their shared evolutionary origin [7].

Transitional species such as *Tiktaalik*, lungfish, and *Polypterus* retain both gills and lungs, serving as extant models of bimodal respiration that bridge aquatic and terrestrial life [8,9,10]. The transition to land required major physiological innovations—including greater pulmonary compliance, expanded vascularization, and more efficient surfactant production—alongside the co-evolution of endocrine pathways and glucocorticoid signaling, which became essential for developmental regulation [4,11,12].

**Figure 3:**
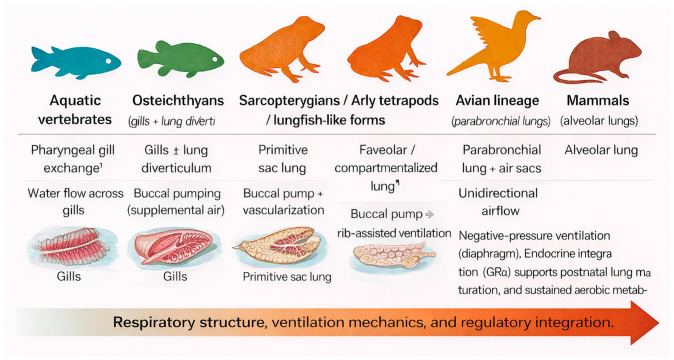
**Evolutionary Transition from Gills to Lungs in Vertebrates**. **Legend**: This figure illustrates the major stages of vertebrate respiratory evolution, tracing the transition from aquatic, gill-based gas exchange to the highly specialized alveolar lungs of mammals. Early jawless vertebrates relied on pharyngeal slits primarily for ion regulation and acid–base balance rather than efficient oxygen uptake. In ancestral bony fishes (Osteichthyes), an unpaired pulmonary diverticulum arose from the foregut, enabling facultative air breathing under hypoxic conditions. Transitional forms such as *Tiktaalik* possessed both functional gills and vascularized lungs, representing a key stage of bimodal respiration during the water-to-land transition. Amphibians and early tetrapods evolved compartmentalized lungs with increased surface area and partial airflow compartmentalization, while birds further refined this design into a parabronchial lung system with air sacs that support continuous, unidirectional gas exchange. Mammals completed this evolutionary trajectory with highly alveolated lungs and the development of a diaphragm, enabling sustained high-volume ventilation and increased metabolic endurance. Collectively, this structural progression established the evolutionary framework within which glucocorticoid receptor α (GRα) signaling emerged as a unifying regulatory system, integrating oxygen sensing, vascular development, surfactant production, and systemic stress adaptation across vertebrate evolution with partial unidirectional airflow, a system that birds later refined into parabronchial lungs with air sacs supporting continuous gas exchange. Mammals completed this evolutionary sequence with highly alveolated lungs and a diaphragm, enabling continuous high-volume ventilation and enhanced metabolic endurance. This structural progression established the evolutionary framework within which GRα signaling emerged as a unifying regulatory system—integrating oxygen sensing, vascular development, surfactant synthesis, and systemic stress adaptation across vertebrate evolution (*the authors acknowledge Chat GPT 5.2’s assistance in creating this figure*).

Figure 3 illustrates the key milestones in vertebrate respiratory evolution, showing the progressive transition from gill- to lung-based respiration and the growing integration of GRα signaling into pulmonary physiology [1,4,8,11,13].

Table 1 (Evolutionary Progression of Vertebrate Respiratory Systems and Associated Corticoid Signaling Functions) complements this overview by summarizing structural, physiological, and endocrine innovations that occurred across vertebrate lineages and their relevance to the emergence and refinement of GRα-centered respiratory homeostasis.

Across lineages, birds evolved rigid parabronchial lungs with air sacs that enable unidirectional airflow, while crocodilians independently developed a similar mechanism—a striking case of convergent evolution [14,15]. In mammals, the emergence of highly alveolated lungs and diaphragm-driven negative-pressure breathing enabled continuous, high-volume ventilation and supported greater aerobic metabolic capacity [8,13]. Together, these structural refinements laid the developmental and physiological groundwork for embryonic lung formation and the later incorporation of GRα-signaling in respiratory adaptation.

### 2.3. Embryological Specification of the Respiratory Tract

The respiratory system begins as a ventral outpouching of the foregut endoderm, forming the laryngotracheal diverticulum. Proper separation from the esophagus is essential; when this process fails, tracheoesophageal fistulas may develop [8,16]. Development of the respiratory tract requires continuous epithelial–mesenchymal communication and is regulated by several key molecular pathways—including Wingless/Integrated (Wnt), Fibroblast Growth Factor (FGF), Bone Morphogenetic Protein (BMP), and Sonic Hedgehog (SHH)—which act together to direct airway branching and lung morphogenesis.

Wnt/β-catenin signaling establishes the proximal–distal axis of the developing lung, determining which regions form conducting airways and which become gas-exchanging structures. Wnt also activates FGF10 and BMP4, linking these pathways into a unified regulatory network [17,18]. FGF10, produced by surrounding mesenchyme, stimulates airway bud outgrowth and epithelial branching, whereas FGF9 helps define distal lung identity [19,20]. BMP4, concentrated at the distal tips of developing airways, fine-tunes branching morphogenesis and promotes differentiation of alveolar type II cells that produce surfactant [21]. SHH signaling delineates the tracheoesophageal boundary and regulates mesenchymal proliferation, thereby maintaining airway structural integrity.

Together, these pathways coordinate branching morphogenesis—the iterative subdivision of airway buds that forms the bronchial tree—and establish the regional patterning that specifies tracheal, bronchial, and alveolar lineages. In late fetal life, GRα-signaling becomes essential for terminal lung maturation: it upregulates surfactant synthesis, promotes alveolar fluid clearance, and prepares the lungs for effective post-natal air breathing (Figure 2) [22,23]. Recent high-resolution imaging and single-cell transcriptomic analyses have revealed the precise temporal and spatial coordination of these developmental signals, clarifying how they regulate the epithelial lineage commitment, airway branching, and alveolar differentiation during mammalian lung formation [24]. Importantly, many of these developmental pathways are re-engaged during lung repair and regeneration in adulthood, underscoring their lifelong contribution to respiratory homeostasis.

### 2.4. Evolution of Specialized Respiratory Cells

Building on the structural transition from gills to lungs—an evolutionary process extensively characterized by Liem—the cellular and molecular diversification of the mammalian respiratory system represents a second significant evolutionary refinement [5]. This diversification did not arise de novo, but through the progressive modification and repurposing of ancestral respiratory programs. Specialized respiratory cells evolved through both innovation and repurposing of ancestral mechanisms, transforming ancient gill-based programs into complex networks of alveolar, vascular, and sensory cells optimized for terrestrial oxygen requirements and the high metabolic demands of endothermy.

Evolutionary Origins and Cellular Specialization. Single-cell transcriptomic studies reveal that the mammalian alveolar capillary network contains two major endothelial populations with distinct functions. This specialization represents a defining evolutionary innovation in mammalian lung design. Aerocytes (aCap cells) are a mammal-specific endothelial subtype specialized for gas exchange and immune surveillance. In contrast, general capillary (gCap) cells regulate capillary perfusion and function as progenitor cells that support ongoing endothelial maintenance and repair. This dual specialization, absent in reptiles and other non-mammalian vertebrates, represents an evolutionary innovation that significantly improved gas-transfer efficiency while strengthening local immune defense [25,26,27,28].

Comparative analyses across mammals, reptiles, and birds show that while core gene-expression programs in alveolar type I (AT1) and type II (AT2) cells are conserved, mammals developed additional intracellular signaling modules and distinct ultrastructural adaptations that sustain endothermy and support the continuous, high oxygen demand of aerobic metabolism [26,29,30]. These refinements reflect selective pressure not only for efficient gas exchange, but also for metabolic resilience under sustained aerobic load.

Hypoxia-Sensitive Cells and Ancestral Repurposing. Two principal oxygen-sensing cell types—pulmonary neuroendocrine cells (PNECs) and carotid-body glomus cells—illustrate how ancient oxygen-sensing mechanisms were adaptively repurposed during vertebrate evolution. These cells likely derive from neuroepithelial oxygen-sensing programs originally present in fish gills. In mammals, PNECs (endoderm-derived) serve as environmental sentinels within the airway epithelium, whereas glomus cells (neural-crest-derived) monitor arterial oxygen tension. This evolutionary transition from externally located to internally integrated oxygen sensing represented a critical step toward precise physiological self-regulation in air-breathing vertebrates [31,32,33,34].

Regeneration and Developmental Plasticity. Beyond their role in gas exchange, gCap cells serve as endothelial progenitors essential for vascular repair. Following injury, a transient population of stem-like endothelial cells regenerates both gCap and aerocyte populations, restoring capillary integrity and function [27,28]. This regenerative capacity underscores the dynamic, rather than static, nature of the alveolar microvasculature.

Endothelial specification depends on tightly regulated signaling networks and developmentally programmed alternative-splicing events, particularly around birth. These mechanisms support the rapid transition to air breathing and ensure optimal postnatal gas-exchange efficiency [26,35,36].

Developmental Conservation and Biomechanical Innovation. These evolutionary refinements are recapitulated during embryogenesis, where conserved signaling pathways—including Wnt, FGF, Notch, and GRα-dependent glucocorticoid signaling—govern the emergence of specialized respiratory subtypes [24,35]. This recurrence highlights deep conservation of developmental logic, even as structural complexity increased.

The mammalian bronchial tree and acinar architecture are optimized for space-filling branching geometry, minimal diffusion distance, and maximal gas-exchange surface area—principles that trace back to the primitive vascular transport systems of early metazoans [37].

Complementing these structural refinements, the evolution of the muscular diaphragm introduced a major biomechanical innovation. Negative-pressure ventilation enabled high-volume tidal breathing, sustained aerobic metabolism, and decoupling of respiration from locomotion—forming the physiological foundation of endothermy [5].

These structural and biochemical innovations occurred in parallel with the progressive evolutionary specialization of GR signaling from its ancestral corticoid receptor. As developed further in Section 2, this molecular coevolution provided an endocrine framework that integrated respiratory development with metabolic and stress adaptation, culminating in coordinated lung maturation, surfactant production, and oxygen homeostasis (Figure 2).

### 2.5. Cellular Crosstalk and Structural Complexity

Building on both evolutionary adaptations and developmental processes, the mature respiratory system exhibits remarkable cellular heterogeneity and functional plasticity, supporting lifelong capacities for defense, adaptation, and repair. Advances in single-cell RNA sequencing (scRNA-seq) and lineage-tracing approaches demonstrated that respiratory cell populations engage in continuous, bidirectional communication across the lifespan, actively remodeling tissue architecture and function in response to metabolic needs and environmental challenges [38,39,40].

The airway and alveolar epithelia comprise multiple specialized cell types—secretory, ciliated, basal, and ionocyte populations—each contributing uniquely to barrier integrity, environmental sensing, and immune coordination. Disruption of epithelial–mesenchymal signaling or imbalances within epithelial cell subpopulations underlie many chronic and infectious respiratory diseases [39,41]. Beyond the epithelium, mesenchymal, immune, vascular, and neuroendocrine cells display substantial phenotypic diversity and exhibit context-dependent reprogramming, highlighting the adaptive versatility of the respiratory microenvironment [42,43].

Single-cell and lineage-tracing studies have further demonstrated that distinct stem and progenitor populations sustain homeostasis and orchestrate regeneration following injury. Epithelial cells can undergo lineage transitions, while fibroblasts and endothelial cells dynamically modulate their activation state in response to stress, exemplifying multi-lineage plasticity that underlies effective tissue repair [39,44]. Together, these stem and progenitor populations display context-dependent plasticity that supports both routine homeostasis and effective regeneration after injury [38].

Cellular crosstalk among epithelial, mesenchymal, endothelial, and immune lineages is essential for coordinating lung growth, the physiologic transition to air breathing at birth, and post-injury repair. The respiratory immune network is spatially compartmentalized, with distinct programs in the upper versus lower airways—an evolutionary adaptation that protects delicate gas-exchange surfaces while maintaining mucosal tolerance [42]. Finally, integrative control extends to the neural level: specialized serotonergic neuron subtypes within the brainstem are developmentally programmed to regulate distinct components of respiratory rhythm, chemosensitivity, and ventilatory drive [45].

### 2.6. Summary and Evolutionary Significance

From its origins in aquatic respiration to the highly specialized alveolated lungs of mammals, the respiratory system exemplifies an evolutionary continuum shaped by rising energy demands and the transition to terrestrial life. Structural adaptation and repurposing of ancestral gills, integrated with conserved developmental signaling networks, enabled the emergence of specialized epithelial, endothelial, and neuroendocrine lineages that sustain oxygen homeostasis and metabolic resilience. These same pathways, refined through evolution, continue to govern lung growth, postnatal adaptation, and regenerative repair throughout life. The mature respiratory system’s cellular heterogeneity and dynamic crosstalk reflect the culmination of these evolutionary and developmental processes—forming an integrative design that unites efficient gas exchange with lifelong adaptability, robust immune defense, and ongoing structural renewal. Figure 1 summarizes the major cellular and structural innovations underpinning this evolutionary progression, from ancestral gill-based respiration to the specialized alveolar architecture of mammals. Understanding these evolutionary principles provides a unifying framework for interpreting congenital lung disorders, improving the management of acute and chronic respiratory diseases, and guiding strategies to restore respiratory homeostasis across the spectrum of critical illness [4,7,12,13,38].

The evolutionary innovations that enabled efficient oxygen uptake and metabolic stability also required the emergence of equally sophisticated regulatory systems to coordinate stress responses, energy distribution, and tissue maturation. Among these, the GRα signaling network evolved as a central integrative mechanism linking environmental stress to cellular and systemic adaptation. Its progressive specialization closely paralleled the evolution of the vertebrate respiratory apparatus, aligning oxygen sensing, metabolic regulation, and developmental control into a unified framework that sustains homeostasis across diverse environmental and physiological transitions.

## 3. The Evolution of Glucocorticoid Signaling and Its Role in Shaping Vertebrate Respiratory Adaptation

The GRα, a ligand-activated transcription factor, evolved early in vertebrates from a common ancestral corticoid receptor shared with the mineralocorticoid receptor (MR). Gene duplication and subsequent sequence divergence produced a receptor with increasingly selective sensitivity to glucocorticoids, enabling more precise regulation of stress adaptation, energy balance, and oxygen homeostasis [46,47,48].

Throughout vertebrate evolution, GRα signaling became increasingly integrated with hypoxia-sensing pathways, particularly the hypoxia-inducible factor 1-alpha (HIF-1α) system. This bidirectional crosstalk allows GR to modulate HIF-1–dependent transcription under hypoxic conditions, while hypoxia and HIF-1α activity reciprocally influence GRα’s regulatory capacity. GR activation can stabilize HIF-1α by suppressing the Von Hippel–Lindau (pVHL) complex, further linking stress-response and oxygen-sensing networks [49,50,51,52].

The emergence of this integrated GR–HIF system was pivotal for the vertebrate transition to terrestrial life, providing a molecular framework that coupled environmental stress detection to coordinated metabolic, immune, and respiratory adaptation. Building on these evolutionary foundations, subsequent sections explore how glucocorticoid signaling continues to coordinate respiratory development, immune regulation, and metabolic resilience. This integrative framework is depicted in Figure 2, which traces the emergence and specialization of GR signaling in parallel with vertebrate respiratory adaptation.

### 3.1. Evolutionary Emergence of GR and Its Functional Integration

The GR evolved from an ancestral corticoid receptor shared with the mineralocorticoid receptor (MR) through gene duplication and structural diversification, enabling selective responsiveness to glucocorticoid and more precise regulation of stress adaptation and metabolic control [46,47,53]. This molecular innovation coincided with the vertebrate transition from aquatic to terrestrial life, when tight regulation of inflammation, vascular tone, and surfactant synthesis became critical for efficient gas exchange and survival in fluctuating oxygen environments [54,55]. Over evolutionary time, permissive mutations, receptor-domain specialization, and the emergence of species-specific isoforms increased the sensitivity and functional breadth of glucocorticoid signaling, deepening GRα’s integration with endocrine, immune, and respiratory pathways [48,56,57]. Collectively, these evolutionary advances established GRα as a central regulatory axis capable of sustaining homeostasis during metabolic demand, inflammatory challenge, and environmental stress [54,55].

In addition to its evolutionary emergence, GRα expression and regulatory dominance are dynamically modulated across developmental stages and physiological contexts, with increasing functional relevance during late gestation, the perinatal transition, and stress adaptation in postnatal life. Unlike the mineralocorticoid receptor and other steroid receptors, GRα exhibits broad tissue distribution and context-dependent regulation through ligand availability, receptor isoform expression, co-regulator interactions, and signaling crosstalk, enabling both steroid-dependent and ligand-independent actions (see Section 3 and Section 4 and Table 2).

### 3.2. GR Regulation of Lung Development: Evidence from Genetic Models

The GR is indispensable for lung maturation and perinatal adaptation in mammals. Global GR knockout mice die at birth from respiratory failure, exhibiting marked structural immaturity, impaired alveolar formation, and persistent cellular proliferation [58,59]. These mice also fail to develop adrenal chromaffin cells, resulting in the absence of the critical catecholamine surge required for lung fluid clearance, surfactant release, and cardiovascular stabilization at birth [58,60]. Collectively, these findings demonstrate that GR coordinates pulmonary, endocrine, and vascular maturation—integrating respiratory, metabolic, and hemodynamic transitions essential for successful extrauterine life.

Conditional knockout models have clarified the tissue-specific mechanisms. Loss of GR in lung mesenchymal cells reproduces the global knockout phenotype, confirming that mesenchymal GR is critical for epithelial–mesenchymal crosstalk and proper alveolar differentiation [23,59,61]. Mesenchymal GRα limits excessive cell proliferation, regulates fibroblast differentiation, and supports extracellular matrix (ECM) organization, including elastin and versican synthesis—both required for alveolar septation [62,63]. By coordinating growth and differentiation programs across epithelial, mesenchymal, and vascular compartments, GRα ensures synchronized morphogenesis and functional readiness of the perinatal lung.

### 3.3. Molecular Pathways Under GR Control in Developing Lung

GR orchestrates key molecular programs that coordinate cell proliferation, ECM remodeling, and surfactant synthesis during lung development. GRα suppresses midkine (Mdk), a pro-proliferative growth factor, while inducing p21^CIP1, a cyclin-dependent kinase inhibitor that halts cell division and promotes differentiation [64,65]. In parallel, GRα represses versican (Vcan)—a large chondroitin sulfate proteoglycan that drives mesenchymal expansion—and induces ADAMTS12, a protease mediating Vcan turnover, thereby maintaining balanced ECM remodeling and promoting alveolar septation [62,64,65]. Together, these coordinated actions ensure controlled mesenchymal growth, proper elastin deposition, and orderly alveolar septation—processes that are disrupted when GRα is absent.

At the epithelial interface, GR directly enhances transcription of surfactant proteins (SP-A, SP-B) and the ABCA3 lipid transporter, functionally preparing alveolar type II cells for efficient gas exchange and lamellar body formation [66,67,68,69]. This ABCA3–surfactant module is illustrated in Figure 2. GR expression peaks late in gestation, coinciding with the fetal cortisol surge and the onset of surfactant production, marking a critical window of glucocorticoid sensitivity required for perinatal respiratory readiness [68,70].

Beyond its critical developmental functions, GRα expression persists across multiple pulmonary cell types, providing ongoing coordination among structural, vascular, immune, and metabolic processes that maintain respiratory homeostasis (Table 2, Representative Cell Populations Expressing GRα Across the Respiratory System and Their Principal Regulatory Functions Supporting Pulmonary Homeostasis) [22,67,69,71,72,73,74,75,76,77,78,79,80,81,82,83,84,85,86]. This broad distribution across pulmonary cell types underscores GRα’s ongoing function as an integrative regulator coordinating epithelial, mesenchymal, endothelial, and immune pathways essential for lifelong respiratory homeostasis.

**Table 2:** Representative Cell Populations Expressing GRα Across the Respiratory System and Their Principal Regulatory Functions Supporting Pulmonary Homeostasis.

Cell/Structure	GRα Expression	Principal GRα-Supported Functions for Pulmonary Homeostasis
Ciliated airway epithelial cells	Yes	Supports epithelial differentiation and mucociliary clearance programs; coordinates anti-inflammatory transcriptional responses with NF-κB [22,87,88].
Goblet/secretory cells	Indirect regulation	Modulates mucin dynamics and epithelial defense during infection/inflammation [72,89].
Conducting airway epithelium (bulk)	Yes	GR–NF-κB cooperation to repress inflammatory genes; clock-dependent control of epithelial inflammatory tone [88,90,91].
Alveolar type II (AT2) cells	Yes	Induces/coordinates surfactant system (SP-B, SP-C, SP-A, ABCA3); tunes mRNA stability and promoter activity [92,93,94,95,96,97].
Alveolar type I (AT1) cells	Yes (lower than AT2, context-dependent)	Barrier support and maturation; tight-junction integrity and permeability control [34,98,99].
Alveolar epithelium (barrier function, general)	Yes	Rapid/non-genomic ion transport effects relevant to fluid balance; circadian gating of GC action on inflammation [90,100,101].
Pulmonary endothelial cells	Yes	Vascular homeostasis; Wnt/β-catenin restraint; angiogenesis control; regeneration programs after injury [27,73,74,102,103].
Airway smooth muscle (ASM)	Yes	Anti-proliferative/anti-hypertrophic signaling; synergism with β2-agonists; modulation of EGFR signaling and transcriptome.
Mesenchyme/fibroblasts & ECM	Yes (developmental and adult)	Guides mesenchymal differentiation for morphogenesis; regulates ECM remodeling and fibroblast activation programs [14,63,104,105,106].
Alveolar macrophages (AM)	Yes	GR-mediated repression of inflammatory pathways (e.g., p38 MAPK); gene-specific repression of inflammatory programs [107,108,109].
Interstitial macrophages (IM)	Yes	Nerve-associated/regulatory IM subsets; antigen handling and local immune regulation under homeostasis [110,111,112,113].
Lung epithelial cells (antigen presentation, immune orchestration)	Yes	Antigen presentation to shape CD4^+^ TRM and barrier immunity; integration with UPR/AP pathways during stress [114,115]
Lymphatic endothelial cells (LECs)	Yes (contextual evidence)	Immune tolerance/antigen scavenging; PD-L1–mediated T-cell regulation; lymphatic function in resolution of inflammation/ARDS [116,117,118,119]
Respiratory microbiome–epithelium interface *	—	GR programs intersect with epithelial–microbiome crosstalk shaping local immunity and homeostasis [120,121,122]
Developmental lung compartments (summary)	Widespread (cell-type specific)	GR controls branching/maturation and cell-lineage decisions; GR loss disrupts epithelial proportions and viability [69,123,124,125].

**Legend**: Glucocorticoid receptor alpha (GRα) is expressed across epithelial, endothelial, mesenchymal, and immune compartments of the lung, integrating hormonal, metabolic, and inflammatory cues to sustain respiratory homeostasis. Through coordinated genomic and non-genomic mechanisms, GRα promotes epithelial differentiation and surfactant synthesis, strengthens endothelial and barrier integrity, modulates airway tone, regulates extracellular-matrix remodeling, and reprograms macrophage inflammatory activity. In lymphatic and stromal networks, GRα signaling contributes to immune tolerance and efficient resolution of inflammation. Collectively, these actions preserve gas-exchange efficiency, prevent fibrosis, and maintain pulmonary stability during development, adaptation, and stress. * The interface row summarizes processes at the mucosal surface rather than a single cell type. **Abbreviations**: GRα, glucocorticoid receptor alpha; AM, alveolar macrophage; AP-1, activator protein-1; ASM, airway smooth muscle; AT1/AT2, alveolar type I/type II epithelial cells; β2-AR, beta2-adrenergic receptor; ECM, extracellular matrix; EGFR, epidermal growth factor receptor; GILZ, glucocorticoid-induced leucine zipper; HIF, hypoxia-inducible factor; IM, interstitial macrophage; LEC, lymphatic endothelial cell; MAPK, mitogen-activated protein kinase; NF-κB, nuclear factor kappa-light-chain-enhancer of activated B cells; PD-L1, programmed death-ligand 1; SP-A/B/C, surfactant proteins A, B, and C; TGF-β, transforming growth factor beta; TLR, Toll-like receptor; TRM, tissue-resident memory T cell; UPR, unfolded protein response; Wnt, Wingless-related integration-site signaling pathway.

Collectively, these findings demonstrate how GRα integrates structural maturation with endocrine timing to ensure a seamless transition from fetal to postnatal respiration. This coordinated developmental strategy not only enables survival at birth but also reflects an evolutionary mechanism in which hormonal signaling anticipates physiologic demands, orchestrates organ maturation, and adapts respiratory function to environmental change. This organizing principle is further developed in the next section.

### 3.4. Evolutionary and Translational Implications

The indispensability of GRα for perinatal respiratory adaptation reflects strong evolutionary selection for endocrine programs that anticipate parturition and proactively prepare the fetus for the abrupt transition to air breathing. Clinically, this same principle underlies the use of antenatal glucocorticoids to patients at risk of preterm delivery, which accelerates the final maturation steps necessary for effective pulmonary gas exchange [22,67,69].

Recent evidence underscores the need for greater precision in translating this evolutionary principle to clinical care. Findings from both animal and human studies demonstrate that therapeutic efficacy depends critically on the timing, dosage, and appropriate patient selection, emphasizing the importance of defined gestational-age windows, dosing limits, and the avoidance of non-indicated exposure. Emerging data on long-term neurodevelopmental vulnerability further highlight the importance of aligning treatment with GRα’s intrinsic developmental timing and tissue-specific sensitivity [23,126,127]. Moreover, dysregulation of GRα signaling during critical developmental windows may imprint enduring susceptibility to chronic respiratory diseases such as asthma and COPD—reinforcing the importance of balancing short-term perinatal gains with consideration of lifelong outcomes [128].

#### Section Summary and Translational Implications

In summary, GRα exemplifies an evolutionary innovation that bridges endocrine signaling with respiratory adaptation. The emergence of glucocorticoid signaling represents a pivotal turning point in vertebrate evolution, enabling the development of complex respiratory structures required for terrestrial life. In the mammalian lung, GRα regulates proliferation, differentiation, and tissue architecture through coordinated mesenchymal–epithelial crosstalk and precise transcriptional control. Its evolutionary conservation and proven clinical relevance underscore GRα’s central role not only in developmental physiology but also in preserving and restoring respiratory homeostasis across acute, chronic, and critical illness.

## 4. Glucocorticoid Receptors in Fetal Lung Development and Respiratory Function

GC signaling through the GRα is essential for the proper development and maturation of the fetal respiratory tract. This pathway regulates a broad spectrum of cellular processes, including mesenchymal and epithelial cell differentiation, progenitor proliferation, ECM remodeling, and surfactant production—laying the foundation for effective postnatal respiration. In the following section, we explore how GRα integrates these diverse developmental programs into a coordinated regulatory network that prepares the lung for birth and supports the abrupt transition to extrauterine life.

### 4.1. Mesenchymal Differentiation and Epithelial–Mesenchymal Crosstalk

GRa signaling within pulmonary mesenchymal cells is indispensable for the differentiation of proliferative mesenchymal progenitors into matrix-producing fibroblasts and for orchestrating epithelial–mesenchymal crosstalk during lung development. This differentiation process coordinates mesenchymal maturation with ECM remodeling and activation of signaling pathways—including VEGF, JAK-STAT, and WNT—that guide alveolar epithelial progenitors toward mature type I (AT1) and type II (AT2) lineages [23,63,125].

Targeted deletion of GRα in mesenchymal cells prevents the transition of mesenchymal progenitors into matrix fibroblasts, resulting in defective ECM-gene expression (*Fn1*, *Col16a4*, *Eln*), excessive proliferation of SOX9^+^ epithelial progenitors, and failure of AT1/AT2 maturation [23,61,124]. These defects highlight the non-cell-autonomous regulatory influence of mesenchymal GRα on epithelial differentiation and lung morphogenesis.

By coordinating matrix remodeling and paracrine signaling (see Section 2.3), mesenchymal GRα ensures balanced growth and proper alveolar septation [61,62]. Conversely, deletion of GRα in epithelial or endothelial compartments has relatively minimal structural effect, underscoring the dominant regulatory role of mesenchymal GRα in coordinating epithelial differentiation through paracrine signaling [63,123]. This compartment-specific hierarchy indicates that mesenchymal GRα functions as a central organizer of developmental signaling, integrating extracellular matrix dynamics with epithelial lineage progression.

Together, these GRα-driven programs integrate matrix remodeling with growth-factor balance to ensure coordinated mesenchymal differentiation and epithelial maturation [23,129].

### 4.2. Transcriptional Regulation and Surfactant Synthesis

Glucocorticoids modulate gene expression in the developing lung by activating the GR, which directly binds to DNA and recruits coactivators to regulate key transcriptional programs. GR activation induces expression of transcriptional regulators such as Hif3a and Zbtb16, which are essential in cytoskeletal organization and epithelial progenitor maturation during fetal lung development [130].

GC-GR signaling also promotes autophagy and surfactant synthesis by upregulating critical autophagy-related genes, including *Becn1*, *Atg7*, and *Lc3b*. These genes facilitate lamellar body biogenesis, enabling the proper storage and secretion of surfactant proteins at birth. Recent studies have shown that the CCAAT/enhancer-binding proteins (C/EBPs)—a family of basic leucine-zipper transcription factors (notably C/EBPα and C/EBPβ)—act as key partners of GR, binding to CCAAT motifs in surfactant-gene promoters and coordinating terminal differentiation of alveolar type II cells. C/EBPs cooperate with GR to recruit steroid receptor coactivators SRC-1 and SRC-2, thereby amplifying transcriptional responses essential for surfactant production and alveolar maturation [131].

Moreover, SRC-1/2 double-deficient fetal mice exhibit reduced expression of 11β-hydroxysteroid dehydrogenase type 1 (11β-HSD1), further limiting local GC activation and downstream gene transcription. These findings emphasize that optimal surfactant synthesis depends not only on GR activation but also on the integrity of its coactivator network, underscoring a multilayered regulatory system that prepares the fetal lung for extrauterine life [131,132].

### 4.3. Effects of GR Deficiency by Cell Type

Loss of mesenchymal GR causes severe lung immaturity, characterized by impaired fibroblast and epithelial differentiation and disruption of alveolar septation [59,63]. These findings demonstrate that mesenchymal GR signaling is the primary driver of lung morphogenesis, regulating epithelial–mesenchymal interactions that shape alveolar architecture. In contrast, epithelial-specific GR deletion increases epithelial proliferation and interferes with the maturation of alveolar type I (AT1) cells, while overall lung structure remains largely intact [59,69]. This indicates that epithelial GR primarily supports terminal epithelial differentiation rather than governing broader architectural patterning within the developing lung. Finally, endothelial-specific GR deletion produces minimal developmental abnormalities, suggesting that GR signaling within the pulmonary endothelium plays a comparatively minor role during fetal maturation [59,60].

### 4.4. Broader Developmental and Systemic Roles

Beyond the lung, GR signaling helps coordinate organ development and metabolic preparedness for postnatal life. Within the lung, GR regulates alveolar epithelial composition by promoting differentiation into AT1 cells, which are critical for efficient gas exchange. In GR-null mice, this differentiation is disrupted, leading to a higher proportion of AT2 cells and fewer AT1 cells, thereby compromising respiratory transition at birth. These findings highlight the central role of GR in establishing the cellular structure of the lung and overall readiness for life outside the womb.

As discussed in Section 2.4 (“Evolutionary and Translational Implications”), understanding the developmental timing and tissue specificity of GRα signaling is essential for translating these mechanisms into safe and effective clinical practice. From a translational perspective, clinical management should emphasize gestationally appropriate timing and avoid unnecessary repeat dosing, linking antenatal steroid administration to coordinated neonatal follow-up of neurocognitive and respiratory outcomes. Future research should refine gestational windows of GRα sensitivity, establish clearer dose–response relationships along the lung–brain axis, and develop biomarkers that distinguish beneficial acceleration of maturation from maladaptive developmental programming [22,23,126,128].

#### Developmental Implications

GR signaling functions as a master regulator of fetal lung development, integrating mesenchymal–epithelial communication, ECM remodeling, surfactant synthesis, and vascular coordination to ensure structural and functional readiness for air breathing. Disruption of this pathway leads to profound defects in lung architecture and gas-exchange capacity, compromising neonatal survival and underscoring GR’s indispensable role in fetal transition to extrauterine life. Collectively, these developmental functions illustrate how GRα operates as a unifying endocrine regulator that anticipates physiologic demands, synchronizes organ maturation, and establishes the foundation for lifelong respiratory resilience.

## 5. The Respiratory System: Complexity, Architecture, and Biofunctional Beauty

The respiratory system is an intricately organized, multicellular network in which epithelial, endothelial, immune, and stromal cells operate in precise spatial coordination to support air filtration, gas exchange, and host defense. A defining feature of this system is the near-universal expression of GRα, a nuclear receptor that adjusts gene expression in response to stress, inflammation, and metabolic cues. By integrating signals across cell lineages, GRα enables rapid adaptation to infection, hypoxia, and injury, positioning it as a central regulator of respiratory resilience and homeostasis.

This section outlines the structural and functional organization of the respiratory tract—spanning epithelial diversity, alveolo-capillary design, mucosal immunity, vascular and interstitial networks, respiratory musculature, and gas-exchange regulation—to establish the framework within which GRα operates. Situating GRα within this architectural landscape underscores its essential role as both a mediator of stress responses and a unifying integrator of respiratory physiology.

### 5.1. Anatomical and Functional Overview

Evolved for both survival and adaptation, the human lung is one of biology’s most exquisitely engineered systems—an intricately ordered, multicellular network of extraordinary scale and precision. It is composed of an estimated 40 to 50 billion cells, including more than 11 billion epithelial and over 20 billion endothelial cells, which together establish the structural and functional backbone of respiratory physiology [25,133,134]. Across this cellular landscape, alveolar type I and II epithelial cells, specialized capillary endothelial subsets (aerocytes and gCap cells), ciliated and secretory airway cells, and diverse immune and stromal populations are organized in highly precise spatial networks that enable continuous air filtration, gas exchange, and host defense.

As reviewed in the next section, nearly every cell within this intricate system expresses the GRα. This nuclear receptor not only responds to systemic glucocorticoid signals but also serves as a central integrator of cellular homeostasis, modulating gene expression in response to stress, inflammation, and metabolic demand. Through coordinated, cross-compartment signaling among epithelial, endothelial, immune, and stromal compartments, GRα aligns respiratory function with systemic homeostasis, allowing rapid and efficient adaptation to infection, hypoxia, and injury. This widespread, lineage-spanning expression underscores GRα’s role as a unifying regulatory hub that connects cellular behavior to organism-level physiologic demands.

Together, these interconnected components provide the framework for understanding how GRα coordinates adaptive and protective responses across the diverse cell populations of the lung—a theme explored further in Section 5.2. This architectural overview establishes the anatomical and functional context needed to understand GRα’s integrative role in maintaining respiratory stability across health and disease.

### 5.2. Structural-Functional Integration

The respiratory tract exhibits a finely tuned structural organization in which form and function are tightly interwoven to optimize air conduction and gas exchange. It is generally divided into the conducting zone—comprising the nasal passages, pharynx, larynx, trachea, and bronchi—and the respiratory zone, which includes the bronchioles and alveoli [135]. The conducting tract primarily conditions inspired air by warming, humidifying, and filtering it, while the respiratory zone enables efficient gas exchange across a large alveolar surface lined with type I and type II alveolar epithelial cells [136,137]. These epithelial cells not only facilitate passive diffusion of oxygen and carbon dioxide but also contribute to host defense by secreting pulmonary surfactants, especially SP-A and SP-B, which lower alveolar surface tension and boost antimicrobial activity [72]. Depending on lung inflation status, the total alveolar surface area ranges from approximately 70 to 100 square meters, providing a large interface for gas exchange [133].

The airway epithelium comprises ciliated, goblet, basal, and club cells, which collectively support mucociliary clearance, secrete protective mucins, and maintain epithelial barrier integrity. The mucociliary escalator functions through the coordinated activity of motile cilia and mucus layers to transport inhaled particles toward the oropharynx. MUC5AC and MUC5B, secreted by goblet and submucosal gland cells, respectively, create a biochemical trap that captures pathogens and promotes their removal. This first line of defense is further enhanced by antimicrobial peptides, collectins, and immunoglobulins, providing broad-spectrum innate immune protection. These epithelial and mucosal defenses are dynamically regulated by GRα signaling, which fine-tunes mucin production, strengthens tight-junction integrity, and calibrates cytokine responses to maintain a balanced immune environment [87,88,89].

Beneath the epithelial layer, the alveolo-capillary interface enables efficient gas exchange across a thin barrier composed of type I pneumocytes and pulmonary capillary endothelial cells. Recent research has shown that the capillary endothelium is not a uniform structure but consists of two main specialized endothelial subsets: aerocytes and general capillary (gCap) endothelial cells (Gillich et al., 2020; Schupp et al. 2020) [25,102]. Aerocytes, unique to the lungs, are extremely thin with minimal cytoplasm, reducing the diffusion distance for oxygen and carbon dioxide and thereby optimizing gas exchange. They also support immune surveillance by facilitating leukocyte trafficking across the alveolar-capillary barrier during inflammation [25,138].

In contrast, gCap cells are more abundant and play complementary roles in regulating vascular tone, mediating angiocrine signaling, and supporting endothelial repair [25,27]. These cells function as progenitors that aid in capillary regeneration after injury and communicate locally through paracrine signals with nearby epithelial and immune cells. Additionally, gCap cells release angiocrine factors that shape tissue responses and help maintain lung homeostasis, a role increasingly recognized in single-cell studies [103,134]. The functional compartmentalization between aerocytes and gCap cells highlights the complexity of the alveolar microvasculature and demonstrates how structural specialization supports both efficient gas exchange and adaptive immune responses. As discussed in the next section, these endothelial functions are further regulated by GRα signaling, which coordinates responses to environmental and inflammatory stress.

To further clarify the structure and function of the alveolo-capillary interface, researchers have developed organ-on-chip models and advanced 3D imaging platforms that accurately reproduce the biomechanical and cellular environment of the alveolus. These systems support co-culture of epithelial and endothelial cells, simulate cyclic breathing mechanics, and enable real-time visualization of responses to pathogens, mechanical forces, and therapeutic interventions [139,140]. Such technologies provide powerful insights into epithelial barrier integrity, immune-cell dynamics, and regenerative signaling within the alveolar niche, effectively bridging the gap between in vivo physiology and translational disease modeling.

### 5.3. Pulmonary Vasculature Architecture and Function

The pulmonary vasculature is a complex and extensive network of arteries, capillaries, and veins arranged in sequence, built to support efficient gas exchange and respond to changing ventilation-perfusion requirements. This high-compliance, low-resistance system allows significant increases in blood flow with minimal pressure fluctuations—especially important during physical activity or under hypoxic conditions [141,142].

This extensive capillary network surrounds the alveoli, with each alveolus tightly connected to a dense web of capillaries, ensuring optimal efficiency in gas exchange. The total length of capillaries in the human lung is estimated to range from 2746 km to 6950 km, depending on the stereological assumptions [143]. These vessels are very narrow—about 6–8 μm in diameter—requiring red blood cells to pass in a single file. This configuration increases the surface area of RBC–endothelium contact and optimizes oxygen and carbon dioxide diffusion by minimizing the diffusion distance and maximizing exposure time [25,144].

As discussed in Section 3.2, advanced imaging has revealed that the alveolar capillary network comprises specialized endothelial subsets, including aerocytes and gCap cells, which facilitate gas exchange, modulate vasomotor tone, and coordinate immune signaling [25,27,102]. This structural specialization enables the lung to rapidly adapt to metabolic demands and inflammatory challenges. As detailed in Section 3.3, these processes are further regulated by GRα signaling, which maintains pulmonary vascular homeostasis.

### 5.4. Pulmonary Interstitium: The Lung’s Immuno-Structural Interface

The pulmonary interstitium, once viewed merely as connective scaffolding, is now recognized as a highly dynamic compartment that integrates mechanical support, immune regulation, fluid balance, and tissue repair. It occupies the space between the alveolar epithelium and capillary endothelium, extending into the peribronchial and perivascular regions, and forms a continuous network of extracellular matrix (ECM), interstitial fibroblasts, pericytes, immune cells, and lymphatics. This compartment significantly contributes to lung compliance and elasticity, enabling the parenchyma to deform during respiration while maintaining alveolar integrity.

Extracellular matrix (ECM) and fibroblast dynamics. The ECM—rich in elastin, collagen, proteoglycans, and glycoproteins—is produced and remodeled by interstitial fibroblasts in response to injury, mechanical stress, and cytokine signaling [105,106,145]. This remodeling balances tensile strength with flexibility, enabling repetitive mechanical stretch without structural failure.

Pericytes, immune cells, and lymphatic vessels cooperate with fibroblasts to regulate immune signaling, interstitial fluid clearance, and tissue repair [110,146,147]. By integrating mechanical cues with biochemical signaling, the ECM–fibroblast network maintains tissue stability while permitting adaptive remodeling under stress. Disruption of this regulatory balance promotes pathological fibrosis, edema, and impaired gas exchange [148].

Interstitial immune surveillance and immune quiescence. The lung interstitium serves as a critical immune surveillance zone, hosting resident memory T cells, interstitial macrophages (IMs), dendritic cells, and innate lymphoid cells (ILCs). These immune cells detect pathogens and tissue injury while limiting inflammation that could damage alveolar structures [111,115]. Unlike alveolar immune cells, interstitial immune populations are strategically positioned to regulate inflammation without directly disrupting gas exchange. IMs, distinct from alveolar macrophages, are positioned along alveolar walls and vasculature, where they modulate immune tone through IL-10 and TGF-β production, supporting tissue homeostasis [110,112,113]. This positioning allows IMs to function as sentinels that fine-tune inflammation at the epithelial–vascular interface, limiting collateral injury during immune activation.

Pulmonary lymphatics: fluid balance and immune regulation. The lymphatic network within the pulmonary interstitium is essential for lung fluid balance and immune surveillance. It provides continuous drainage of interstitial fluid, plasma proteins, immune cells, and macromolecules—preventing pulmonary edema and preserving lung compliance [118,147,149].

Beyond fluid homeostasis, pulmonary lymphatics actively regulate immunity by transporting antigens, dendritic cells, and immune mediators to regional lymph nodes, thereby initiating and shaping adaptive immune responses [117,150].

Lymphatic endothelial cells (LECs) are not passive conduits but active immunoregulatory cells. They release cytokines and chemokines, present antigens via MHC molecules, and regulate leukocyte migration through inhibitory molecules, such as PD-L1, which restrain T-cell activation and promote immune tolerance [116,151,152,153].

For CD8^+^ T cells, LECs present peripheral tissue antigens (PTAs) without costimulation, inducing deletion or anergy via MHC-I and PD-L1 signaling [116,152,153]. For CD4^+^ T cells, LECs function as antigen reservoirs, transferring antigens to dendritic cells that promote T-cell anergy or regulatory T-cell differentiation [154].

Microbial sensing and mechanical regulation. Emerging evidence indicates that LECs may respond to signals from the lung microbiota, potentially linking lymphatic function to host-microbe interactions and immune adaptation. Although direct lung-specific data remain limited, LEC expression of pattern-recognition receptors (e.g., TLRs) supports this regulatory potential [155,156].

LECs are sensitive to mechanical cues such as stretch and shear stress, which may regulate lymphangiogenesis and immune signaling under physiological and during mechanical ventilation. Lymphatic remodeling occurs during chronic inflammation, altering drainage capacity and immune regulation. During neonatal transition, pulmonary lymphatics play a vital role in clearing fetal lung fluid, enabling effective air breathing [119,157]. These insights position pulmonary lymphatics as a potentially modifiable therapeutic target across acute and chronic lung diseases.

Disruption of lymphatic drainage or LEC function leads to fluid retention, chronic inflammation, and disease progression in ARDS, COPD, and pulmonary fibrosis—highlighting pulmonary lymphatics as a modifiable therapeutic target across acute and chronic lung disease.

Stromal–immune crosstalk and GRα regulation. Stromal–immune interactions within the lung interstitium are tightly regulated by paracrine signaling from fibroblasts, epithelial cells, vascular endothelium, and immune populations. Stromal cells secrete chemokines (e.g., CCL2, CXCL10), cytokines (e.g., IL-6, IL-1β), and ECM components that shape immune recruitment, activation, and differentiation [158,159,160,161,162,163]. Immune cells reciprocally influence stromal phenotype and function, creating feedback loops that calibrate inflammation and tissue repair [164,165]. This bidirectional signaling ensures immune responsiveness without destabilizing lung architecture.

Glucocorticoid receptor alpha (GRα), as reviewed in Section 5, plays a central role in modulating cytokine production, immune cell recruitment, and tissue repair dynamics. While lung-specific stromal studies remain limited, evidence from non-pulmonary models demonstrates that stromal GRα is essential for glucocorticoid-mediated anti-inflammatory responses. In arthritis models, deletion of GRα in fibroblast-like synoviocytes abolished glucocorticoid efficacy despite intact GRα in immune cells—highlighting the dominant regulatory role of stromal GR [166]. These findings strongly suggest that similar GRα-dependent stromal–immune mechanisms operate within the pulmonary interstitium. In this setting, GRα signaling regulates fibroblast phenotype, ECM dynamics, immune quiescence, and cytokine feedback—particularly by modulating IL-6 family cytokines and TGF-β signaling [81,159].

Through these integrated actions, stromal GRα preserves lung architecture under stress, promotes inflammation resolution, and prevents maladaptive remodeling and fibrosis—positioning it as a central regulator of interstitial homeostasis and a promising therapeutic target in both acute and chronic lung disease [73,82,167].

### 5.5. Immune and Microbial Surveillance

Lung immunity is orchestrated by a complex network of epithelial cells, immune cells, and the resident microbiome that together maintain homeostasis at the air–tissue interface. Epithelial cells act not only as physical barriers but also as active immune sentinels, detecting pathogens through pattern-recognition receptors (PRRs) and initiating antimicrobial and regulatory responses. They secrete cytokines, chemokines, and antimicrobial peptides that recruit and instruct resident and circulating immune cells—such as alveolar macrophages, dendritic cells, neutrophils, and T lymphocytes—and thereby coordinate innate and adaptive immune defenses [120,168,169,170,171,172,173,174].

This epithelial–immune crosstalk is bidirectional: immune cells, in turn, send feedback signals that influence epithelial phenotype, barrier integrity, and inflammatory resolution. Depending on the context, these exchanges promote either immune tolerance or chronic inflammation [114,172,175]. By regulating both activation and resolution programs, epithelial cells help maintain barrier stability and prevent immune-mediated tissue damage [170,171].

The lung microbiome—a dynamic community of commensal microorganisms—adds a third regulatory layer that modulates immune tone, maintains tolerance, and enhances resistance to pathogens [120,122]. Microbial metabolites and cell-wall components interact with epithelial and immune receptors to calibrate inflammatory thresholds and sustain mucosal equilibrium. When this microbial balance is disturbed—by antibiotics, infection, or environmental stressors—immune signaling becomes dysregulated, increasing susceptibility to infection and chronic inflammatory lung diseases [121,176,177].

Together, this tripartite surveillance system—epithelial signaling, immune activation, and microbial modulation—forms the foundation of respiratory immune homeostasis. Disruption of any single component—or of their coordination—can shift the system from balanced immunity to pathological inflammation or impaired defense. Understanding this integrated framework is essential for developing therapeutic strategies that restore tolerance, resolve inflammation, and strengthen mucosal defenses [120,122,175].

### 5.6. Respiratory Musculature and Mechanics

Breathing is driven by the rhythmic contraction of the primary and accessory respiratory muscles, which work together to create the pressure gradients needed for air movement. Under resting conditions, quiet breathing is maintained by the coordinated activity of the diaphragm and external intercostal muscles. In contrast, the accessory muscles—such as the sternocleidomastoid and scalene—are recruited during increased ventilatory demand, including physical exertion, speech, or respiratory distress [178,179,180]. These muscle groups contain a high proportion of oxidative, fatigue-resistant fibers—especially in the diaphragm—allowing them to contract continuously throughout life. However, this endurance comes with a cost: prolonged metabolic stress, systemic inflammation, or increased ventilatory demand can lead to respiratory-muscle fatigue and weakness, which may contribute to respiratory failure in critical illness [181].

Neural regulation of breathing is orchestrated by a distributed brainstem network that integrates chemical and mechanical feedback. Central chemoreceptors located near the ventrolateral medulla detect elevations in arterial CO_2_ (PCO_2_) and accompanying reductions in pH, thereby increasing respiratory motor output to restore gas-exchange homeostasis [182]. The retrotrapezoid nucleus (RTN) serves as a key chemosensory hub within this network, interacting with the pre-Bötzinger complex, which generates the inspiratory rhythm, and pontine-medullary centers that modulate phase transitions between inspiration and expiration. The nucleus of the solitary tract (NTS) integrates afferent input from peripheral chemoreceptors and mechanoreceptors, relaying these signals to spinal motor neurons that drive the diaphragm and intercostal muscles, ensuring coordinated and adaptive respiratory effort [183].

This finely tuned neural control system ensures respiratory muscles respond effectively to changing physiological demands. However, its efficiency can be compromised in pathological conditions where increased mechanical load, inflammation, or disrupted neural signaling progressively reduce muscle performance and coordination. In diseases such as chronic obstructive pulmonary disease (COPD) and neuromuscular disorders, altered motor recruitment patterns, increased load, and reduced endurance capacity weaken respiratory muscle function, leading to impaired ventilation and dyspnea [184].

As described in later sections, GRα signaling opposes these maladaptive processes by maintaining mitochondrial bioenergetics, redox balance, and calcium homeostasis in respiratory and accessory muscles. By sustaining ATP generation, limiting oxidative injury, and stabilizing excitation–contraction coupling, GRα enhances contractile endurance and supports adaptive responses to heightened metabolic and mechanical stress.

Beyond its cell-specific roles summarized in Table 2, GRα orchestrates integrated signaling networks that link the airway, vascular, mesenchymal, immune, and muscular compartments of the lung into a unified regulatory system. Through these interconnected pathways, GRα coordinates cellular metabolism, inflammatory control, and structural maintenance to preserve respiratory stability under stress. Table 3 (Functional Integration of GRα Signaling Across Pulmonary Compartments) outlines the main cross-compartmental actions and key molecular mediators through which GRα maintains pulmonary homeostasis, supports repair, and promotes adaptive resilience across the respiratory system.

### 5.7. Gas Exchange and Developmental Adaptation

Efficient gas exchange depends on a large alveolar surface area, an exceptionally thin diffusion barrier, and accurate ventilation–perfusion (V/Q) matching, all maintained through continuous alveolar and microvascular development. The processes of alveolarization and capillary expansion extend well into postnatal life, improving both gas exchange efficiency and the lung’s capacity for structural repair [133,187]. Oxygen and carbon dioxide passively move across the alveolar–capillary interface, where red blood cells serve as efficient carriers for gas transport. The system’s effectiveness depends on precise ventilation–perfusion (V/Q) matching, which is primarily regulated by hypoxic pulmonary vasoconstriction (HPV), directing blood flow away from poorly ventilated regions toward those with optimal aeration [188]. This adaptive vasomotor response preserves arterial oxygenation during regional lung dysfunction and exemplifies the finely tuned coupling between ventilation, perfusion, and microvascular signaling.

#### Functional Integration of Respiratory Homeostasis

The respiratory system functions as a complex and highly integrated network that coordinates airway architecture, vascular dynamics, immune surveillance, and microbial symbiosis. Its impressive ability to detect, respond, and adapt to environmental and physiological challenges reflects both its evolutionary development and innate resilience. Disruption of any structural or regulatory component—epithelial, immune, vascular, or muscular—can destabilize the entire system, emphasizing its delicate balance and essential integrative function.

Many of these adaptive and homeostatic processes are regulated by the endocrine system, especially glucocorticoids acting through the GRα. GC-GRα signaling influences key respiratory functions—surfactant production, immune response, vascular tone, epithelial repair, and interstitial remodeling—linking molecular signals with tissue-level adaptation. By coordinating these processes across diverse cell populations, GRα provides a unifying regulatory framework that supports structural integrity, metabolic efficiency, and immune balance throughout the respiratory tract.

The broad distribution and cell-specific actions of GRα throughout the respiratory tract are summarized in Table 2, which illustrates how this receptor coordinates structural integrity, immune balance, and functional homeostasis across diverse pulmonary cell types. Recognizing this integrated regulatory system is essential for understanding how the respiratory tract adapts to stress, inflammation, and injury—and for designing targeted therapeutic strategies that restore physiological balance and preserve lung function.

## 6. GC-GRα Signaling Regulation of Lung Function: Integrating Gas Exchange, Barrier Integrity, Fluid Clearance, and Inflammatory Control

Having established the structural and cellular organization of the respiratory system, this section now examines how glucocorticoid (GC)–glucocorticoid receptor alpha (GRα) signaling governs pulmonary function through coordinated molecular and physiological mechanisms. GRα acts as a master transcriptional regulator of respiratory homeostasis, coordinating a variety of processes essential for maintaining efficient lung performance throughout life. Beyond its developmental role in lung maturation, GRα continues to regulate airway reactivity, surfactant synthesis, immune balance, vascular coupling, epithelial integrity, and alveolar fluid clearance. Together, these interconnected actions work to preserve gas exchange efficiency, structural integrity, and adaptability in response to inflammation, oxidative stress, or environmental challenges. This section explores how GRα signaling connects these physiological subsystems, integrating insights from developmental biology, cellular signaling, and translational research.

### 6.1. Glucocorticoid Receptor Regulation of Tracheobronchial Tree and Airway Tone Regulation

GC–GRα signaling in the lung operates through both steroid-dependent mechanisms, including classical genomic and rapid non-genomic actions triggered by ligand binding, and steroid-independent mechanisms mediated by context-dependent signaling cross-talk, protein–protein interactions, and chromatin regulation. The following subsections (Section 6.1, Section 6.2 and Section 6.3) illustrate how these complementary modes integrate to regulate airway tone, surfactant biology, barrier integrity, and inflammatory resolution.

The GC-GRα signaling pathway is crucial for controlling airway smooth muscle (ASM) tone through its combined anti-inflammatory, bronchodilatory, and antiproliferative effects—mechanisms that explain how glucocorticoids work in asthma. When activated, GRα reduces ASM contraction by lowering intracellular calcium, decreasing muscarinic and histamine receptor activity, and inhibiting the inflammatory induction of bradykinin B2 receptors [76,189]. Concurrently, GRα promotes smooth muscle relaxation by increasing β_2_-adrenoceptor expression and stimulating Na^+^/K^+^-ATPase activity, thereby improving membrane repolarization and aiding airway dilation [77,78].

To counter airway hyperresponsiveness, GRα induces MAPK phosphatase-1 (MKP-1/DUSP1), which deactivates pro-contractile MAPK signaling and suppresses ASM proliferation and hypertrophy through KLF15- and PLCD1–dependent transcriptional regulation [78,185,190]. DUSP1 represents the principal acute anti-inflammatory effector downstream of GRα activation, mediating rapid dephosphorylation of p38, JNK, and ERK MAPKs and thereby terminating AP-1– and NF-κB–dependent cytokine amplification. Loss of DUSP1 abrogates glucocorticoid anti-inflammatory efficacy despite intact GRα signaling, establishing this phosphatase as a non-redundant molecular switch that converts glucocorticoid signaling into inflammatory resolution rather than generalized immunosuppression [191,192,193,194]. Additionally, GRα limits cytokine and chemokine production—such as IL-6 and CXCL8—in the airway wall, thereby restoring immune balance and reducing local inflammation. Emerging evidence indicates that airway smooth muscle cells can produce local glucocorticoids and dynamically regulate GR expression, potentially contributing to sustained airway homeostasis and steroid responsiveness [195]. Together, these integrated genomic and non-genomic mechanisms explain the therapeutic effectiveness of glucocorticoids in asthma—keeping the airways open, reducing hyperresponsiveness, and lowering disease severity. The broader genomic and non-genomic anti-inflammatory actions of GC-GRα signaling in the respiratory system, including integration with metabolic and stress-response pathways, are reviewed in detail elsewhere [196].

### 6.2. Glucocorticoid Receptor Regulation of Surfactant Production and Airspace Patency

The GRα is indispensable for preparing the lung for air breathing at birth. It provides gene-specific regulation of pulmonary surfactant homeostasis, especially during late gestation and the early postnatal transition to air breathing. Inside alveolar type II (AT2) epithelial cells, GRα activates coordinated transcriptional networks that promote the production of surfactant proteins (SPs) and lipid transporters, which are needed for alveolar stability and optimal lung compliance after delivery. One of the most critical GRα targets, the ATP-binding cassette transporter A3 (ABCA3), is essential for the formation of lamellar bodies—specialized intracellular organelles that package, store, and secrete surfactant lipids and proteins. Through direct interaction with a glucocorticoid-response element (GRE) in the ABCA3 promoter, GRα upregulates ABCA3 mRNA and protein expression, thereby increasing intracellular surfactant reserves in preparation for postnatal air breathing. This ABCA3–surfactant module is shown in Figure 2. During late gestation, ABCA3 expression increases significantly, ensuring sufficient surfactant production and alveolar patency during the transition from liquid to gas respiration [94].

Surfactant proteins B (SP-B) and C (SP-C), which lower alveolar surface tension and prevent collapse at end-expiration, are also induced by glucocorticoids through GRα-dependent transcriptional activation. SP-B expression increases quickly and independently of new protein synthesis, indicating a primary, direct genomic effect of GRα. Conversely, SP-C induction is delayed and relies on secondary GR-responsive cofactors that facilitate epithelial differentiation and maturation [63,92,93]. Beyond transcriptional control, GRα also enhances SP-B mRNA stability via 3′-UTR–dependent mechanisms, thereby sustaining surfactant protein availability during the critical perinatal window [95].

Surfactant protein A (SP-A) exhibits a distinctive biphasic regulatory pattern—initially increasing, then decreasing after prolonged glucocorticoid exposure. This adaptive transcriptional switch is regulated by GRα-dependent recruitment of histone deacetylase 2 (HDAC2) and associated local chromatin condensation, which together fine-tune SP-A expression and prevent excessive surfactant buildup in the alveolar space [96,97,197].

Recent transcriptomic studies confirm that antenatal glucocorticoid signaling via GRα orchestrates a broad developmental program in the fetal lung—linking surfactant production with lipid metabolism, mitochondrial biogenesis, and antioxidant defenses. This network prepares the lung for the sudden oxidative and mechanical challenges of extrauterine life [63,198,199]. Through these precisely timed GRα-regulated processes, the fetal lung achieves the structural, metabolic, and functional maturity needed for alveolar expansion, effective gas exchange, and continued postnatal breathing. Clinically, this GRα-driven cascade explains the well-known effectiveness of antenatal corticosteroid therapy, which accelerates fetal lung development and greatly reduces the risk and severity of neonatal respiratory distress syndrome (RDS).

### 6.3. Glucocorticoid Receptor Regulation of Alveolar-Capillary Barrier Integrity and Interstitial Homeostasis

In this compartment, GRα function is highly context-dependent, integrating ligand-activated genomic and non-genomic signaling with microenvironmental cues such as inflammation, mechanical stress, and hypoxia to preserve alveolar–capillary barrier integrity and interstitial homeostasis. The alveolar–capillary barrier maintains selective permeability between airspaces and the circulation, ensuring efficient gas exchange while preventing fluid transudation. In acute lung injury (ALI) and ARDS, disruption of this barrier leads to plasma leakage, interstitial and alveolar edema, and severe hypoxemia. GC-GRα signaling acts as a central homeostatic defense system that preserves barrier integrity by reinforcing junctional complexes, reducing cytoskeletal tension, and attenuating inflammation.

Through both genomic and non-genomic actions, GRα stabilizes tight junctions, modulates actomyosin contractility, and reduces inflammatory injury. In experimental models, dexamethasone decreases myosin light-chain kinase (MLCK) and myosin light-chain 2 (MLC2) phosphorylation while increasing zonula occludens-1 (ZO-1) and claudin-8 expression, thereby enhancing epithelial cohesion and limiting tumor necrosis factor-alpha (TNF-α)–induced permeability [98,99,200]. These molecular effects restore barrier selectivity and decrease paracellular fluid leakage under inflammatory conditions [98,200].

Beyond barrier stabilization, GRα promotes epithelial repair and interstitial homeostasis. By interacting with developmental transcription factors such as signal transducer and activator of transcription 3 (STAT3), GRα facilitates alveolar progenitor differentiation and epithelial regeneration following injury [125]. Loss of epithelial GRα impairs junctional structure, enhances cytokine expression, and slows recovery, confirming its dual role in maintaining barrier integrity and orchestrating tissue repair [201,202].

During fetal development, GRα guides the maturation of the alveolar–capillary interface, coordinating epithelial, endothelial, and mesenchymal differentiation. GRα deficiency leads to thickened alveolar septa, decreased lung compliance, and impaired gas exchange, while antenatal corticosteroid therapy activates GR-responsive transcriptional networks that assemble junctional proteins and extracellular matrix components vital for postnatal lung [125,185,203].

Collectively, these findings identify GRα as a key regulator of alveolar integrity and interstitial homeostasis. By coordinating structural, inflammatory, and reparative processes, GRα maintains fluid balance and prevents pulmonary edema—mechanisms that explain the well-known clinical effectiveness of corticosteroid therapy in both acute respiratory distress syndrome (ARDS) and preterm lung maturation.

### 6.4. Glucocorticoid Receptor Regulation of Pulmonary Lymphatics

Glucocorticoid receptors, especially GRα, are essential regulators of lung lymphatic and immune functions, functioning at the intersection of hormonal signaling, circadian rhythms, and immune cell trafficking. In T lymphocytes and other immune cells, GR activation influences their development and effector functions, ultimately affecting lung immune responses [83]. A central anti-inflammatory mechanism involves GR-mediated suppression of IL-1-induced IL-6 production in lung fibroblasts, achieved through both transcriptional repression and post-transcriptional regulation, thereby reducing cytokine-driven inflammation [204].

Beyond direct cytokine regulation, GRs influence the spatial distribution of immune cells through circadian mechanisms. GRα signaling increases interleukin-7 receptor (IL-7R) and C-X-C chemokine receptor type 4 (CXCR4) expression in T cells, promoting their rhythmic migration between the lungs, blood, and lymphoid organs, thereby supporting adaptive immunity in synchrony with the body’s diurnal cycle [84]. In pulmonary epithelial cells, GR binding to the CXCL5 gene exhibits rhythmicity, regulating chemokine production and driving time-of-day-specific neutrophil recruitment. These circadian and endocrine mechanisms work together to coordinate pulmonary immunity, linking GR activation to the local molecular clock that controls leukocyte trafficking and the timing of inflammation [90].

Recent transcriptomic and mechanistic studies further demonstrate that GRα signaling regulates pulmonary vascular and lymphatic permeability through crosstalk with Wnt/β-catenin and nuclear factor-κB (NF-κB) pathways, enhancing endothelial stability and limiting inflammatory leak [73,91]. The function of GRα is fine-tuned by post-translational modifications such as phosphorylation and by co-regulatory proteins, including Merm1 (Mediator of ERBB2-driven cell motility 1), which amplify GRα transcriptional activity and determine steroid responsiveness within inflamed tissues [205,206]. The disruption of these co-regulators—or of circadian integrity—can weaken GRα activity, leading to glucocorticoid resistance and impaired inflammation resolution [207].

At the protein-interaction level, GRα binds with caveolin-1 in pulmonary tissue to regulate the transcription of anti-inflammatory genes; however, the absence of caveolin-1 does not eliminate GRα-mediated inflammatory suppression in vivo, indicating that specific GRα cofactor interactions are modulatory rather than strictly indispensable [208].

Taken together, GRα signaling coordinates multiple layers of pulmonary immunity and lymphatic balance by regulating cytokine production, immune cell localization, vascular tone, and circadian rhythms. These integrated actions form the physiological basis for the strong anti-inflammatory and immunomodulatory effects of glucocorticoids in respiratory diseases, while also explaining how disruptions in GRα co-regulators or circadian rhythms can diminish therapeutic benefits.

### 6.5. Glucocorticoid Receptor Regulation of Alveolar Immune Responses and Inflammatory Signaling

The alveolar compartment acts as a primary immunological interface, continuously exposed to airborne pathogens, allergens, and environmental stressors. Maintaining functional homeostasis at this interface requires precise coordination between host defense and inflammation control. GRα signaling serves as a central regulator of this balance, integrating genomic and non-genomic programs that suppress excessive inflammation, support epithelial and immune-cell repair, and limit collateral tissue injury [55].

At the core of alveolar inflammatory regulation are two transcription factors—nuclear factor kappa B (NF-κB) and activator protein-1 (AP-1)—which drive expression of numerous pro-inflammatory cytokines and chemokines. Their activation in alveolar epithelial cells and macrophages promotes production of IL-8, IL-1β, and TNF-α, leading to neutrophil recruitment and barrier dysfunction [209,210]. GRα counteracts these pathways through multiple complementary mechanisms. It directly tethers to NF-κB and AP-1, interferes with their transcriptional activity, and induces inhibitory phosphatases such as dual-specificity phosphatase 1 (DUSP1). GRα also activates anti-inflammatory mediators, including glucocorticoid-induced leucine zipper (GILZ) and IL-10, converging on suppression of cytokine transcription and promotion of inflammatory resolution [108,109,211]. This layered repression allows inflammation to be restrained without abolishing essential host-defense programs.

Macrophage polarization and immune resolution. In alveolar macrophages, NF-κB activation promotes polarization toward a classically activated, pro-inflammatory (M1-like) phenotype characterized by high cytokine output and tissue injury. GRα signaling opposes this shift and facilitates transition toward an M2-like reparative phenotype. By repressing NF-κB and inhibiting p38 MAPK activity, GRα induces IL-10 expression and promotes macrophage programs associated with tissue repair, efferocytosis, and the resolution of inflammation [107,212]. This phenotypic reprogramming restores immune balance while limiting excessive fibrosis and preserving alveolar architecture.

Recent transcriptomic studies further demonstrate that GR activation in alveolar macrophages induces the release of soluble Toll-like receptor 2 (TLR2), which functions as a decoy receptor to dampen inflammatory signaling [213]. This mechanism provides an additional layer of innate immune modulation that restrains excessive pattern-recognition receptor activation.

Epithelial–immune integration and GRα signaling fidelity. In alveolar epithelial cells, GRα signaling is modulated by extracellular stress-response pathways. Extracellular heat shock protein 70 (HSP70) acting through TLR4, enhances GRα expression and signaling capacity. This interaction amplifies anti-inflammatory and antioxidant programs during epithelial stress.

Context-dependent specificity of GRα signaling is further achieved through receptor phosphorylation and interaction with co-regulatory proteins. GRα partners with Mediator of ERK-activated MAPK1-interacting protein 1 (Merm1), GR-interacting protein 1 (GRIP1), and transcriptional intermediary factor 2 (TIF2), which refine transcriptional accuracy and influence glucocorticoid sensitivity. These co-regulators help determine whether GRα signaling promotes resolution versus resistance in inflamed tissues.

Barrier repair and immune containment. In addition to transcriptional immune regulation, GRα directly supports epithelial barrier repair following inflammatory injury. By reducing myosin light-chain kinase (MLCK) activity and phosphorylation of myosin light chain 2 (MLC2), while increasing expression of tight-junction proteins such as zonula occludens-1 (ZO-1) and claudin-8, GRα restores junctional integrity and limits paracellular fluid leak [98,99]. These structural effects complement GRα’s anti-inflammatory actions, coupling immune resolution with physical barrier restoration. By integrating control of inflammation, macrophage phenotype, epithelial integrity, and transcriptional fidelity, GRα maintains alveolar immune balance and prevents progression from adaptive inflammation to tissue-damaging pathology.

#### Clinical Relevance and Integrative Perspective

Collectively, GRα signaling plays a dual and highly adaptive role in alveolar immunity. It suppresses harmful inflammation through transcriptional interference and phosphatase induction, while simultaneously promoting epithelial repair and macrophage-mediated resolution. These coordinated actions provide a mechanistic foundation for the proven clinical effectiveness of corticosteroids in acute respiratory distress syndrome (ARDS) and severe pneumonia. Importantly, this framework explains why therapeutic benefit depends on preserved GRα signaling capacity and appropriate timing, rather than nonspecific immunosuppression—reinforcing the concept of GRα as a homeostatic regulator rather than a blunt anti-inflammatory agent.

### 6.6. Glucocorticoid Receptor Regulation of Cardiopulmonary Vascular Coupling and Endothelial Homeostasis

Glucocorticoid receptors (GRs) are central regulators of cardiovascular and pulmonary homeostasis, coordinating both genomic and non-genomic signaling pathways across cardiomyocytes, vascular smooth muscle, and endothelial cells. Upon activation by endogenous glucocorticoids, GRα modulates gene networks controlling vascular tone, metabolic efficiency, stress adaptation, and immune balance [80,214]. Genomic GRα signaling governs the transcription of structural, metabolic, and anti-inflammatory genes that are crucial for maintaining vascular and myocardial resilience. In contrast, non-genomic pathways—initiated by membrane-associated GR complexes—trigger rapid calcium fluxes and kinase activation, thereby regulating vascular resistance and myocardial excitability [215,216].

Within vascular tissues, GRα signaling preserves hemodynamic stability and structural integrity through cell-specific actions. In smooth muscle, GRα activation enhances catecholamine responsiveness, supporting rapid vasoconstriction during stress and maintaining perfusion pressure. In endothelial cells, GRα not only upregulates endothelial nitric oxide synthase (eNOS) and nitric oxide (NO) production but also represses Wnt/β-catenin–driven inflammatory and angiogenic signaling, thereby maintaining endothelial quiescence and vascular integrity [73,79,214]. Loss of endothelial GRα amplifies Wnt signaling and autophagy-mediated crosstalk, leading to excessive angiogenesis, vascular leak, and fibrotic remodeling [74]. These dual actions—enhancing contractile responsiveness while restraining inflammation—maintain vascular tone and protect against fibrosis and barrier dysfunction, thereby preserving efficient cardiopulmonary vascular coupling.

In cardiomyocytes, GRα signaling controls calcium handling, mitochondrial function, and sarcomere structure, supporting excitation–contraction coupling and myocardial recovery after stress [216,217]. The dynamic balance between GRα and mineralocorticoid receptor (MR) signaling is pivotal: while GRα provides cardioprotective, anti-inflammatory, and anti-fibrotic effects, unchecked MR activation leads to hypertrophy, fibrosis, and hypertension. Pharmacologic MR antagonists restore GRα dominance and mitigate cardiorenal and age-related vascular dysfunction [218,219].

Circadian and epigenetic mechanisms dynamically regulate GRα activity in the heart and vasculature. GRα signaling follows daily cortisol rhythms, aligning vascular tone, cardiac excitability, and metabolic flow with the sleep–wake cycle. The CLOCK–BMAL1 complex, a key part of the molecular circadian clock, controls rhythmic gene transcription and interacts directly with GRα to adjust its cortisol responsiveness over 24 h. Through these interactions, GRα synchronizes cardiovascular and metabolic functions with systemic circadian cues, supporting adaptive performance across different physiological states. Epigenetic modifications—including chromatin remodeling and DNA methylation—further fine-tune GRα sensitivity and gene expression, maintaining the alignment of vascular tone and cardiac excitability with environmental and metabolic demands.

In the pulmonary vasculature, GRα signaling plays a crucial role in maintaining endothelial barrier integrity during inflammation. Activation of GRα within lung endothelial cells strengthens tight-junction complexes, reduces vascular permeability, and protects the alveolar–capillary interface from inflammatory injury [75]. Conversely, diminished GRα activity during systemic stress—such as endotoxemia or sepsis—leads to endothelial destabilization, vascular leak, pulmonary edema, and impaired gas exchange. These coordinated mechanisms show how GRα signaling protects both systemic and pulmonary vascular health. By coordinating transcriptional regulation, vascular reactivity, barrier integrity, and anti-inflammatory control, GRα serves as a central hub of cardiopulmonary homeostasis and a key therapeutic target in vascular and inflammatory disease.

### 6.7. Glucocorticoid Receptor Regulation of Mitochondrial Function and Oxidative Balance

GRα directly regulates mitochondrial metabolism and redox homeostasis, coordinating cellular energy production with antioxidant defense to preserve tissue resilience under stress. This regulatory role is particularly critical in the lung, where continuous exposure to oxygen, mechanical stretch, and inflammation imposes high oxidative and bioenergetic demands.

In pulmonary tissue, GRα activation upregulates antioxidant enzymes—most notably glutathione peroxidase 3 (GPx3)—through direct binding to glucocorticoid-response elements (GREs) within the GPx3 promoter [220]. This transcriptional control enhances pulmonary defense against reactive oxygen species (ROS) and limits oxidative injury to epithelial and endothelial cells. Although initially demonstrated in lung-derived and tumor cell models, accumulating evidence supports a comparable physiological role for GRα-mediated redox regulation in normal respiratory epithelium.

Mitochondrial localization and genomic control. Beyond nuclear transcriptional effects, GRα exerts direct actions within mitochondria. Following activation, GRα can translocate into mitochondria and bind mitochondrial DNA at GRE-like sequences. Through this interaction, GRα regulates transcription of genes encoding oxidative phosphorylation (OXPHOS) complexes, mitochondrial ribosomal proteins, and metabolic enzymes [199,221,222,223].

GRα also interacts with mitochondrial proteins such as Bcl-2 and pyruvate dehydrogenase (PDH), linking hormonal signaling to apoptosis control, calcium handling, and the metabolic shift from glycolysis toward OXPHOS. Mitochondrial import of GRα is facilitated by chaperones including Hsp70, Hsp90, Bag-1, and FKBP51, enabling context-dependent mitochondrial signaling that aligns energy production with stress adaptation [224]. These mechanisms position GRα as a direct regulator of mitochondrial gene expression rather than a purely nuclear stress-response factor.

Dose- and time-dependent metabolic effects. The metabolic effects of glucocorticoids follow a biphasic pattern. Acute or physiological GRα activation enhances mitochondrial respiration, membrane potential, and cell survival. In contrast, chronic or supraphysiologic glucocorticoid exposure promotes oxidative stress, mitochondrial depolarization, and apoptosis [225,226]. This dose- and time-dependence explains both the therapeutic effectiveness of glucocorticoids in acute stress and inflammation and the well-recognized toxicity associated with prolonged exposure. Importantly, these divergent outcomes reflect regulatory imbalance rather than intrinsic mitochondrial toxicity of GRα signaling.

*GR isoforms and mitochondrial specialization*. Distinct GR isoforms further diversify mitochondrial outcomes. While GRα supports oxidative balance and antioxidant defense, GRγ promotes mitochondrial biogenesis and ATP synthesis even in the absence of ligand binding, reflecting specialized, non-redundant control of mitochondrial energy metabolism [227,228,229]. These isoform-specific actions highlight non-redundant control of mitochondrial energy metabolism within the glucocorticoid receptor family.

Tissue-specific expression and alternative splicing of GR transcripts fine-tune glucocorticoid sensitivity and determine cell-type–specific metabolic responses. This regulatory diversity allows mitochondria to adapt energy production to local functional demands across pulmonary epithelial, endothelial, immune, and muscular compartments.

Pathophysiological implications of mitochondrial GR dysregulation. Dysregulated mitochondrial GR signaling contributes to a broad spectrum of disease states, including cancer, immune dysfunction, neurodegeneration, and critical illness. In cancer models, mitochondrial GR overexpression promotes glycolytic reprogramming and increased tumor aggressiveness, whereas in immune cells it alters mitochondrial metabolism and contributes to glucocorticoid resistance [224,230]. In chronic stress states and metabolic disorders, disrupted GR–mitochondrial interactions impair oxidative balance, ATP synthesis, and cellular resilience [231,232].

Collectively, these findings establish GRα as a central regulator of mitochondrial efficiency and redox homeostasis. Physiological activation maintains energy balance and antioxidant capacity, whereas sustained dysregulation leads to oxidative stress, mitochondrial dysfunction, and loss of adaptive reserve.

Integration with epithelial and barrier function. By sustaining mitochondrial ATP generation and redox balance, GRα provides the energetic foundation necessary for high-demand epithelial processes. These mitochondrial actions directly support the ion transport, junctional maintenance, and fluid-handling mechanisms examined in Section 6.1, where GRα governs airway hydration and postnatal alveolar fluid clearance. This energetic coupling links mitochondrial regulation to the epithelial barrier and fluid-control pathways examined in the following section, where GRα governs airway hydration and postnatal alveolar fluid clearance as part of an integrated homeostatic program.

### 6.8. Glucocorticoid Receptor Signaling in Airway Hydration

Glucocorticoid receptors (GRs), especially the GRα isoform, are crucial regulators of airway surface liquid (ASL) homeostasis and alveolar fluid clearance. Through both genomic and non-genomic mechanisms, GR signaling maintains epithelial barrier integrity, ion transport, and the regulation of inflammation. In the airway epithelium, GRα activation increases claudin-8 expression, a tight-junction protein that prevents paracellular sodium leak and enhances epithelial cohesion, thereby helping maintain ASL volume. GRα also upregulates the expression of epithelial sodium channels (ENaC) and aquaporins, facilitating sodium and water absorption, which are vital for postnatal alveolar fluid clearance [233].

Conversely, GR signaling inhibits chloride-secreting pathways by downregulating CFTR (cystic fibrosis transmembrane conductance regulator) and NKCC1 (a sodium–potassium–chloride co-transporter), shifting the lung from fluid secretion to absorption—a transition essential during fetal-to-neonatal adaptation [233]. Beyond ion transport, GR suppresses pro-inflammatory cytokine production and activates anti-inflammatory genes, such as dual-specificity phosphatase 1 (DUSP1), which inhibits MAPK signaling, including through the HSP70-TLR4 axis—a pathway connecting heat-shock proteins and innate immune regulation [234].

Rapid, non-genomic GR actions further regulate airway hydration by rapidly inhibiting chloride secretion through modulation of K channels and secondary messenger pathways, independent of transcriptional activation [100,101]. These mechanisms complement traditional GR-dependent gene regulation to maintain balanced ion transport and ASL composition.

GR function in the airway epithelium is also influenced by isoform expression and nuclear transport efficiency. While GRα mediates canonical glucocorticoid responses, GRβ can antagonize GRα or independently modulate cell migration, apoptosis, and cytokine expression, thereby shaping steroid resistance in airway disease [206,235,236]. Impaired nuclear import via importin-13 (IPO13) or reduced availability of co-regulators such as GRIP1 further limits GRα’s transcriptional efficacy and anti-inflammatory potency [237,238].

By maintaining airway hydration and epithelial stability, GRα preserves distal airway patency and efficient gas exchange. Section 6.2 then extends this framework to the systemic level, detailing how GRα regulates oxygen delivery and carbon dioxide removal.

### 6.9. Glucocorticoid Receptor Regulation of Oxygenation and CO_2_ Homeostasis

GRs integrate hormonal stress responses with cellular oxygen sensing and mitochondrial energy metabolism, thereby coordinating the regulation of oxygen uptake and carbon dioxide (CO_2_) elimination. GR signaling dynamically adjusts tissue metabolism and ventilation to match fluctuating oxygen availability during inflammation, hypoxia, or critical illness.

At the cellular level, GRs engage in bidirectional crosstalk with hypoxia-inducible factors (HIFs)—key regulators of oxygen sensing and adaptation. Ligand-activated GR interacts with HIF-1α and its cofactors to refine gene programs that control angiogenesis, glycolysis, and mitochondrial respiration. Depending on cell type and inflammatory or metabolic state, GR can suppress excessive HIF activation to prevent harmful angiogenesis or enhance HIF-dependent pathways to support tissue survival during hypoxia. This balance is crucial for maintaining vascular integrity, metabolic flexibility, and efficient oxygen delivery in inflammatory, ischemic, or high-altitude environments [49,51].

Beyond its nuclear role, GR translocates to mitochondria, where it binds mitochondrial DNA at GRE-like elements and regulates genes encoding oxidative-phosphorylation (OXPHOS) complexes and metabolic enzymes [199,226]. Through interactions with Bcl-2, pyruvate dehydrogenase, and other mitochondrial partners, GR stabilizes the membrane potential, reduces reactive oxygen species (ROS) formation, and coordinates aerobic metabolism with efficient CO_2_ production. These mitochondrial actions integrate endocrine signaling with cellular bioenergetic demand, enhancing tissue oxygen utilization and redox balance. When GR–mitochondrial communication is disrupted, oxidative efficiency declines, resulting in hypoxemia, hypercapnia, and cellular energy failure—hallmarks of severe systemic inflammation and shock.

At the systemic level, GR maintains feedback regulation of the hypothalamic–pituitary–adrenal (HPA) axis, adjusting circulating glucocorticoid rhythms in response to hypoxia or hypercapnia. This endocrine feedback ensures adaptive respiratory drive, vascular tone, and substrate mobilization, aligning cellular respiration with organ-level gas exchange. By synchronizing metabolic rate, vascular reactivity, and ventilatory demand, GR signaling preserves acid–base balance and supports efficient CO_2_ removal [54].

Collectively, these mechanisms establish GR as a key integrator of oxygen sensing, mitochondrial energy conversion, and ventilatory control. In parallel, GR signaling is essential for stress erythropoiesis—the rapid production of erythroid progenitors in response to anemia, hypoxia, or hemorrhage. Ligand-activated GR promotes the self-renewal of burst-forming-unit-erythroid (BFU-E) cells and their differentiation into colony-forming-unit-erythroid (CFU-E) cells, working together with erythropoietin receptor (EPO-R), c-Kit, and peroxisome proliferator-activated receptor-α (PPARα) pathways to restore oxygen-carrying capacity [239,240]. This hematopoietic action ensures sufficient red-blood cell output and hemoglobin availability to sustain systemic oxygen transport and CO_2_ removal under stress conditions. Clinically, these same GR-dependent mechanisms underlie the use of glucocorticoids to stimulate erythropoiesis in glucocorticoid-responsive anemias, such as Diamond-Blackfan anemia, when erythropoietin alone is inadequate [240].

By enhancing mitochondrial efficiency, preventing HIF overactivation, and stabilizing systemic glucocorticoid rhythms, GR signaling maintains optimal oxygenation and CO_2_ clearance. Additionally, GR-dependent stimulation of erythroid progenitor expansion under hypoxic stress ensures sufficient oxygen-carrying capacity and systemic delivery, indirectly supporting whole-body O_2_/CO_2_ balance [239,240]. In acute respiratory illness, therapeutic activation of GR re-activates these adaptive pathways—restoring mitochondrial function, restraining inflammation, and improving gas-exchange efficiency [51,80,199].

By coordinating mitochondrial metabolism, oxygen utilization, and erythropoietic adaptation, GRα ensures that oxygen delivery and CO_2_ clearance remain aligned with physiological demand. These systemic mechanisms set the stage for the next level of regulation: GRα’s modulation of neural and hormonal pathways that govern ventilatory responses to hypoxia. Section 6.3 examines this higher-order control of respiratory drive and its critical role during environmental or pathological oxygen stress.

### 6.10. Glucocorticoid Receptor Modulation of Hypoxic Ventilatory Response

GR signaling exerts multilevel control over respiratory adaptation to hypoxia, coordinating neural, hormonal, and transcriptional pathways that modulate the hypoxic ventilatory response (HVR). Through these mechanisms, GR signaling fine-tunes respiratory drive, stabilizes gas exchange, and shapes individual variability in ventilatory adaptation.

Experimental studies in neonatal rodents indicate that pharmacological blockade of GR—such as with mifepristone (RU-486), a mixed glucocorticoid and progesterone receptor antagonist—augments HVR by increasing tidal volume during hypoxic exposure. This elevation primarily occurs in males, whereas females naturally exhibit a higher baseline ventilatory response, suggesting that sex-specific GR signaling shapes sex-specific central respiratory response circuits. Similar sex-linked sensitivity has been confirmed using selective GR antagonists (e.g., CORT113176), further underscoring the importance of hormonal and developmental context in respiratory control [241].

At the molecular level, GR signaling intersects with the hypoxia-inducible factor (HIF) pathway, creating a bidirectional regulatory axis that adjusts transcriptional programs for metabolism, angiogenesis, and stress resistance under low-oxygen conditions. Ligand-activated GR can either inhibit or promote HIF-1α-mediated transcription depending on cell type and metabolic state, thus maintaining redox balance while preventing maladaptive vascular or inflammatory responses [51,242]. Conceptually, this interaction aligns with the GR–HIF module illustrated in Figure 2.

Hypoxia can reciprocally influence GR signaling: in some cell types, it upregulates GR expression and enhances anti-inflammatory activity, while in others (e.g., airway epithelium), it inhibits GR nuclear translocation and decreases glucocorticoid sensitivity [80,243]. This context-dependent regulation helps explain why glucocorticoid effectiveness varies across conditions characterized by oxygen stress—including neonatal hypoventilation, pulmonary hypertension, and acute mountain sickness.

Clinically, GR antagonism or dysregulation influences not only ventilation but also stress-hormone release, insulin sensitivity, and immune cell trafficking during hypoxia, highlighting a broader systemic impact. Targeted modulation of GR pathways could therefore enhance ventilatory stability and mitigate inflammatory injury in hypoxia-related conditions [51,244].

The sequential activation of GRα-dependent programs described in Section 5.1, Section 5.2, Section 5.3, Section 5.4, Section 5.5, Section 5.6, Section 5.7, Section 6.1, Section 6.2 and Section 6.3 follows a conserved temporal pattern (Table 4 Phase-Specific Roles of GRα in Pulmonary Homeostatic Correction). During acute stress, adaptation, and recovery, GRα functions through distinct yet overlapping phases that align with the Priming, Modulatory, and Restorative stages of pulmonary homeostatic correction (reviewed in Reference [55]). Together, these dynamic phase-specific actions define GRα as the central coordinator of homeostatic correction in the respiratory system, integrating inflammatory control, metabolic adaptation, and structural repair to restore efficient gas exchange and systemic stability. These phase-specific actions demonstrate how GRα integrates inflammatory control, metabolic adaptation, and structural repair to restore respiratory stability. Section 6 now turns to the molecular architecture of this system—detailing the regulatory mechanisms that shape GRα availability, nuclear translocation, transcriptional specificity, and therapeutic responsiveness.

**Figure 4:**
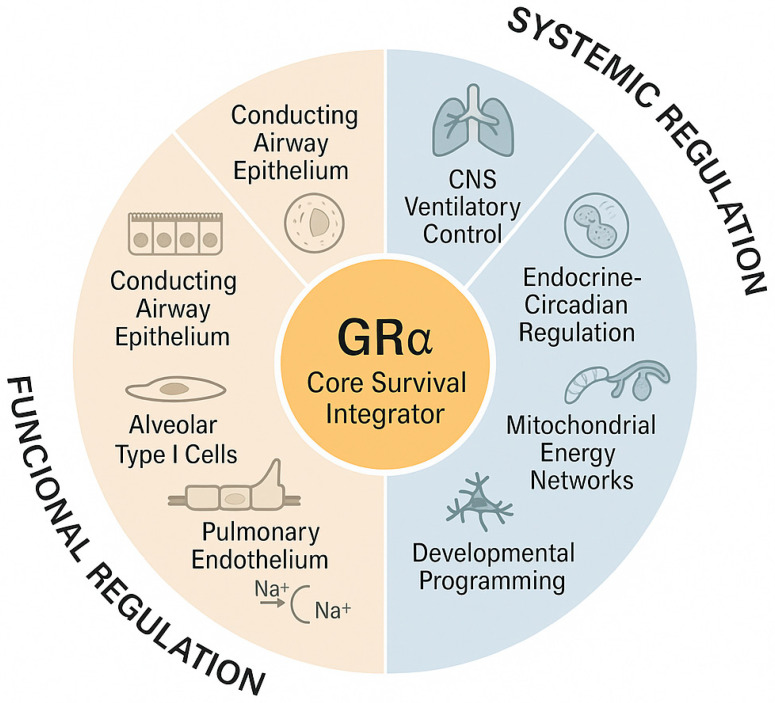
**Central Role of GRα in Coordinating Pulmonary and Whole-Body Physiology**. **Legend**: Glucocorticoid receptor alpha (GRα) functions as a core survival integrator, coordinating pulmonary functional modules with systemic regulatory networks essential for respiratory homeostasis. Within the lung, GRα synchronizes epithelial, endothelial, and ion transport programs that maintain barrier integrity, gas exchange, and fluid balance. At the systemic level, GRα links pulmonary function to ventilatory control, endocrine–circadian signaling, mitochondrial energy supply, neuro-immune integration, and developmental programming, thereby supporting adaptation and survival across physiological states (*the authors acknowledge ChatGPT 5.2’s assistance in creating this figure*).

The system-level interactions illustrated in Figure 4 position GRα as a core integrator of pulmonary homeostasis. Across airway, alveolar, endothelial, immune, neural, and metabolic domains, GRα coordinates rapid protective responses with sustained epithelial repair, vascular stability, and adaptive energy allocation. This integrated framework provides the mechanistic foundation for the concluding section, which examines how these coordinated actions underpin glucocorticoid clinical effectiveness and why preserving—or restoring—GRα function is increasingly recognized as critical in severe and critical illness.

Taken together, the mechanisms outlined across Section 3, Section 4 and Section 5 reveal a unifying principle: glucocorticoid receptor alpha (GRα) does not regulate isolated pulmonary functions, but instead coordinates interdependent structural, metabolic, vascular, immune, and bioenergetic programs required for respiratory stability. Through synchronized control of mitochondrial energy supply, epithelial barrier integrity, immune resolution, fluid clearance, and vascular coupling, GRα enables the lung to adapt dynamically to developmental transitions, circadian variation, environmental stress, and inflammatory injury. This system-level integration provides the mechanistic foundation for both the physiological indispensability of GRα and the context-dependent clinical efficacy of glucocorticoids in respiratory disease—setting the stage for the concluding synthesis that follows.

## 7. Conclusions—Glucocorticoid Receptor Regulation of the Pulmonary System

The pulmonary system sustains life through its remarkable balance between openness and protection—regulating gas exchange, immune defense, fluid balance, and environmental sensing across a vast yet delicate surface. Its structural precision, shaped by the tracheobronchial tree, alveolar epithelium, vascular endothelium, resident immune cells, lymphatic networks, and the diaphragm, requires continuous adjustment to meet physiological challenges while avoiding injury. Central to this coordination is the GR—especially its GRα isoform—which equips the lung to respond effectively to developmental transitions, circadian fluctuations, infectious exposures, and physiological stress.

Through its integrated genomic and non-genomic mechanisms, the GC–GRα signaling system protects the structural, metabolic, and immune integrity of the respiratory tract. It regulates airway tone, surfactant production, epithelial repair, endothelial stability, and interstitial remodeling, all while suppressing excessive inflammation. By transcriptionally regulating cytokines, adhesion and junctional proteins, ion channels, and antioxidant systems, GRα establishes a molecular framework that couples tissue protection to precisely tuned immune responses. Beyond inflammation control, GRα coordinates pulmonary metabolism, mitochondrial efficiency, and circadian immune rhythms, thereby synchronizing repair, host defense, and energy allocation during periods of heightened physiological vulnerability.

At the mitochondrial-hypoxia-inducible interface, GRα functions as a metabolic and oxygen-sensor, maintaining redox balance, optimizing oxidative phosphorylation, and supporting efficient oxygen use. Its actions connect endocrine and cellular adaptation, linking ventilatory control to bioenergetic demand and harmonizing systemic and cellular homeostasis. Although the role of GRα in pulmonary lymphatic endothelium remains incompletely understood, its established regulation of vascular permeability, immune-cell trafficking, and cytokine–lymphatic signaling strongly suggests a unifying role throughout the pulmonary compartments.

This integrative capacity explains the enduring therapeutic effectiveness of glucocorticoids across the respiratory spectrum—from fetal lung maturation and neonatal adaptation to acute respiratory distress syndrome (ARDS), severe pneumonia, and chronic inflammatory lung disease—and points toward a future of precise GR modulation. In clinical practice, this means that glucocorticoid efficacy depends not only on dosing strategies but also on the underlying condition of the GRα signaling system itself. Complementary strategies that maintain or restore GRα number and function—including targeted micronutrient repletion, mitochondrial and antioxidant support, probiotic modulation of the gut–lung axis, and circadian rhythm alignment—serve as essential partners to pharmacologic therapy. Collectively, these approaches stabilize GRα signaling, enhance receptor sensitivity, and shift glucocorticoid treatment from a symptom-directed practice to one focused on restoring homeostatic regulation and strengthening physiological resilience.

Finally, while this review focuses on the respiratory tract, GRα regulates every organ system—including the vascular, lymphatic, neural, immune, metabolic, and neuroendocrine networks—and is indispensable for life [246]. This reconceptualization of GRα as a core survival receptor, essential across development and adult physiology, calls for a fundamental paradigm shift in translational medicine. GRα is not merely a mediator of stress but the central integrative axis of organismal survival, embedded within a broader web of nutritional, microbial, and metabolic systems that collectively govern energy balance, redox homeostasis, tissue integrity, and adaptive resilience. Viewed through this system’s lens, GRα functions as a biological counterpart to the so-called “God particle”—a unifying integrator whose dysfunction destabilizes the coordinated physiologic regulation required for life.

## Figures and Tables

**Table 1:** Evolutionary Progression of Vertebrate Respiratory Systems and Associated Corticoid Signaling Functions.

Evolutionary Stage	Dominant Respiratory Structure	Oxygen Strategy/Efficiency	Key Endocrine Integration	Representative Species	Functional Relevance to GRα Evolution
Primitive aquatic vertebrates	Pharyngeal slits (gills)	Counter-current exchange; ion and acid–base regulation	CR (ancestral corticoid receptor)	Lampreys, hagfish	Ion balance and early stress response signaling [1,2,3]
Early bony fish (Osteichthyes)	Gills + unpaired lung diverticulum	Dual respiration; emergence of air-breathing capacity	Partial CR/GR signaling; early corticosteroids	*Polypterus*, *Lepidosiren*	Initiation of glucocorticoid-mediated oxygen control [4,5,6]
Transitional forms (*Tiktaalik*)	Gills and vascularized lungs	Bimodal respiration; buccal-pump ventilation	Divergence of GR and MR; early GR–HIF1α cross-talk	*Tiktaalik* *roseae*	Establishment of oxygen-dependent GR regulation [8,9,10]
Amphibians and early tetrapods	Paired, compartmentalized lungs	Improved compliance; partial unidirectional flow	Functional HPA axis; GR–MR co-activation	Frogs, salamanders	GR-regulated surfactant synthesis and vascular remodeling [4,11,12]
Birds	Parabronchial lungs with air sacs	Continuous unidirectional airflow; maximal efficiency	GR–HIF1α coordination for oxygen homeostasis	*Gallus gallus*	GR control of erythropoiesis and metabolic resilience [14,15]
Mammals	Alveolated lungs with diaphragm	Continuous negative-pressure ventilation; high metabolic demand	Fully integrated GRα system regulating surfactant, ECM, and vascular integrity	*Mus musculus*, *Homosapiens*	Complete systemic integration of GRα in oxygen and stress homeostasis [8,13]

**Legend**: This table summarizes the evolutionary continuum from aquatic to terrestrial respiration, emphasizing the parallel refinement of GRα-regulated homeostatic control. As vertebrates transitioned from gill-based to lung-based gas exchange, the ancestral corticoid receptor (CR) diverged into glucocorticoid (GR) and mineralocorticoid (MR) receptors, enabling coordinated regulation of ion transport, surfactant synthesis, vascular remodeling, and metabolic adaptation. These integrative mechanisms culminated in the mammalian GRα network, which unites endocrine, immune, and mitochondrial pathways to sustain pulmonary and systemic stability. **Abbreviations**: CR, corticoid receptor; GRα, glucocorticoid receptor alpha; MR, mineralocorticoid receptor; HIF, hypoxia-inducible factor; ECM, extracellular matrix; HPA, hypothalamic–pituitary–adrenal axis.

**Table 3:** Functional Integration of GRα Signaling Across Pulmonary Compartments.

Pulmonary Compartment	Principal GRα Actions	Key Molecular Mediators	Functional Outcome
Airway epithelium [69,88,92,108]	Induces surfactant system; restores epithelial tight junctions; represses NF-κB and AP-1 pathways	SP-B, SP-C, ABCA3, claudin-8, occludin, DUSP1, GILZ	Enhanced mucosal defense and epithelial repair
Pulmonary endothelium [27,73,74]	Restrains Wnt/β-catenin activation; maintains eNOS–NO signaling; limits vascular leak	GR–Wnt interaction, eNOS, caveolin-1, DUSP1,	Preserved endothelial integrity and reduced edema.
Airway and vascular smooth muscles [77,108,185,186]	Reduces hyperresponsiveness; enhances β2-adrenergic responsiveness; limits EGFR-driven remodeling	β2-AR, KLF15, DUSP1, MAPK pathway	Bronchodilation and controlled vascular tone.
Mesenchyme fibroblast [63,105,106]	Modulates TGF-β–Smad axis; limits collagen I/III synthesis; promotes matrix turnover	Versican, IL-6, TGF-β signaling axis, DUSP1, PPARγ	Controlled ECM remodeling and anti-fibrotic balance
Alveolar and interstitial macrophages [107,108,109]	Represses pro-inflammatory genes; induces GILZ and MKP-1; drives resolution programs	GILZ, DUSP1, MAPK dephosphorylation, IL-10	Inflammatory resolution and tissue protection
Lymphatic endothelium [116,117,119]	Promotes immune tolerance and antigen presentation and immune regulation; coordinates fluid drainage	PD-L1, FOXC2, VEGFR-3	Resolution of inflammation and edema prevention
Respiratory muscles and mitochondria [86,108]	Supports oxidative phosphorylation, redox balance, and ATP generation	PGC-1α, KLF9, mt-GR, SOD2	Sustained ventilatory capacity and metabolic resilience

**Legend**: Functional Integration of GRα Signaling Across Pulmonary Compartments. GRα integrates endocrine, metabolic, and immune cues across all major pulmonary compartments. Within the airway epithelium, it induces surfactant synthesis and restores junctional integrity; in the endothelium, it restrains Wnt/β-catenin signaling and preserves vascular barrier function. GRα limits smooth-muscle hyperreactivity, modulates fibroblast activation and extracellular-matrix turnover, and reprograms macrophage responses toward resolution. In lymphatic and stromal networks, GRα promotes immune tolerance and fluid drainage, while in respiratory muscles and mitochondria it sustains oxidative phosphorylation and redox balance. These concerted actions maintain gas-exchange efficiency, reduce fibrosis, and uphold pulmonary homeostasis during development, adaptation, and stress recovery. **Abbreviations**: GRα, glucocorticoid receptor alpha; ABCA3, ATP-binding cassette subfamily A member 3; AP-1, activator protein-1; β2-AR, beta2-adrenergic receptor; DUSP1, dual-specificity phosphatase 1; ECM, extracellular matrix; EGFR, epidermal growth factor receptor; eNOS, endothelial nitric oxide synthase; FOXC2, forkhead box C2; GILZ, glucocorticoid-induced leucine zipper; IL-6/IL-10, interleukins 6 and 10; KLF9/15, Krüppel-like factors 9 and 15; MAPK, mitogen-activated protein kinase; MKP-1, MAPK phosphatase-1; NF-κB, nuclear factor kappa-light-chain-enhancer of activated B cells; NO, nitric oxide; PD-L1, programmed death-ligand 1; PGC-1α, peroxisome proliferator-activated receptor-gamma coactivator 1α; PPARγ, peroxisome proliferator-activated receptor gamma; SOD2, superoxide dismutase 2; SP-A/B/C, surfactant proteins A, B, and C; TGF-β, transforming growth factor beta; VEGFR-3, vascular endothelial growth factor receptor 3; Wnt, Wingless-related integration-site signaling pathway.

**Table 4:** Phase-Specific Roles of GRα in Pulmonary Homeostatic Correction.

Homeostatic Phase	Dominant Pulmonary Targets	Core GRα Functions	Key Mediators	Representative Outcomes
**Priming phase**	Airway epithelium, macrophages	Activates innate defense, surfactant synthesis, and cytokine control	NF-κB repression, SP-B/SP-C, ABCA3	Rapid containment of injury and restoration of gas exchange
**Modulatory phase**	Endothelium, fibroblasts	Limits inflammation, stabilizes vascular barrier, and regulates ECM turnover, and reprograms immunometabolism to restrain inflammatory signaling	GILZ, DUSP1, itaconate (TCA-cycle–derived), TGF-β inhibition	Reduced permeability, anti-fibrotic remodeling
**Restorative phase**	Macrophages, epithelial and muscle cells	Promotes resolution of inflammation, mitochondrial recovery, and tissue repair	PGC-1α, IL-10, Annexin 1	Structural recovery, endurance restoration, restored homeostasis

**Legend**: Across the Priming, Modulatory, and Restorative phases of homeostatic correction, GRα dynamically re-balances immune, vascular, and metabolic activity. During the Priming phase, GRα supports immediate host defense and surfactant induction; in the Modulatory phase, it restrains inflammation in part through immunometabolic reprogramming that supports TCA-cycle–derived itaconate production [245], and maintains endothelial–stromal integrity; and in the Restorative phase, it promotes mitochondrial recovery, resolution of inflammation, and structural repair, ensuring full restoration of respiratory homeostasis. **Abbreviations**: GRα, glucocorticoid receptor alpha; ABCA3, ATP-binding cassette subfamily A member 3; DUSP1, dual-specificity phosphatase 1; ECM, extracellular matrix; GILZ, glucocorticoid-induced leucine zipper; IL-10, interleukin 10; NF-κB, nuclear factor kappa-light-chain-enhancer of activated B cells; PGC-1α, peroxisome proliferator-activated receptor-γ coactivator 1α; SP-B/SP-C, surfactant proteins B and C; TGF-β, transforming growth factor β.

## Data Availability

The original contributions presented in this study are included in the article. Further inquiries can be directed to the corresponding author.

## Abbreviations

The following abbreviations are used in this manuscript:

GRα	glucocorticoid receptor alpha
MR	mineralocorticoid receptor
CR	corticoid receptor
HIF1α	hypoxia-inducible factor 1 alpha
SP-A	surfactant protein A
ABCA3	ATP-binding cassette subfamily A member 3
ECM	extracellular matrix
O_2_	oxygen
